# Overcoming resistance to immune checkpoint therapy in PTEN-null prostate cancer by intermittent anti-PI3Kα/β/δ treatment

**DOI:** 10.1038/s41467-021-27833-0

**Published:** 2022-01-10

**Authors:** Zhi Qi, Zihan Xu, Liuzhen Zhang, Yongkang Zou, Jinping Li, Wenyu Yan, Cheng Li, Ningshu Liu, Hong Wu

**Affiliations:** 1 The MOE Key Laboratory of Cell Proliferation and Differentiation, School of Life Sciences, Peking University, Beijing, China; 2 School of Life Sciences, Peking University, Beijing, China; 3 Peking-Tsinghua Center for Life Sciences, Peking University, Beijing, 100871 China; 4 Institute for Cancer Research, Shenzhen Bay Laboratory, Shenzhen, 518107 China; 5 Bayer AG, Drug Discovery TRG Oncology, Muellerstrasse 178, 13353 Berlin, Germany; 6 Present Address: Hehlius Biotech, Inc., 1801 Hongmei Rd, Shanghai, 200233 China

**Keywords:** Cancer immunotherapy, Targeted therapies

## Abstract

Combining immune checkpoint therapy (ICT) and targeted therapy holds great promises for broad and long-lasting anti-cancer therapies. However, combining ICT with anti-PI3K inhibitors have been challenging because the multifaceted effects of PI3K on both cancer cells and immune cells within the tumor microenvironment. Here we find that intermittent but not daily dosing of a PI3Kα/β/δ inhibitor, BAY1082439, on *Pten*-null prostate cancer models could overcome ICT resistance and unleash CD8^+^ T cell-dependent anti-tumor immunity in vivo. Mechanistically, BAY1082439 converts cancer cell-intrinsic immune-suppression to immune-stimulation by promoting IFNα/IFNγ pathway activation, β2-microglubin expression and CXCL10/CCL5 secretion. With its preferential regulatory T cell inhibition activity, BAY1082439 promotes clonal expansion of tumor-associated CD8^+^ T cells, most likely via tertiary lymphoid structures. Once primed, tumors remain T cell-inflamed, become responsive to anti-PD-1 therapy and have durable therapeutic effect. Our data suggest that intermittent PI3K inhibition can alleviate *Pten*-null cancer cell-intrinsic immunosuppressive activity and turn “cold” tumors into T cell-inflamed ones, paving the way for successful ICT.

## Abstract (web summary)

Limited response to immune checkpoint inhibitors has been reported in patients with castration-resistant prostate cancer. Here the authors show that intermittent, but not continuous, treatment with an anti-PI3Kα/β/δ-inhibitor promotes anti-tumor immunity and response to PD-1 blockade in a preclinical *Pten*-null model of prostate cancer.

## Introduction

Immune checkpoint therapies (ICT), such as those mediated by anti-PD-1 or CTLA-4 antibodies, have shown promising long-lasting effects on certain cancer types by activating T cell-mediated anti-tumor immunity^1^. The efficacy of ICT is positively correlated with the density of tumor infiltrating CD8^+^ T cells^2–4^. However, the majority of solid cancers have poor CD8^+^ T cell infiltration (“cold” tumors) and do not respond to ICT^5^. Although the mechanisms underlying cancer-mediated T cell exclusion are largely unknown, it has become clear that promoting T cell infiltration may increase the range of cancers sensitive to ICT^5,6^.

Prostate cancer is the most common malignancy in males, and the second leading cause of male cancer-related death in the Western world^7^. Androgen deprivation therapy (ADT) is the mainstream treatment for prostate cancer. However, despite initial regression, many patients progress to highly aggressive castration-resistant prostate cancer (CRPC), a disease stage with limited treatment options^8^. Recent ICT trials on CRPC patients have shown disappointing results^9–11^, most likely due to low mutational load and defects in T cell-mediated anti-tumor immunity. It has been reported that over 90% of prostate cancers are “cold” and do not express a T cell-inflamed gene signature^12^. Therefore, treatments that can promote T cell infiltration may pave the way for efficient ICT on prostate cancers.

Loss of the *PTEN* tumor suppressor or activation of its controlled PI3K pathway are associated with resistance to ICT in multiple tumor types^3,13^. In prostate cancer, *PTEN* mutations have been found in 40–50% primary and 70–90% metastatic tumors^14^. PTEN loss in the murine prostatic epithelial in the *PB-Cre*^*+*^*Pten*^*loxP/loxP*^ (*Pten*-null) mouse model mimics both molecular and pathological features associated with human prostate cancers, including upregulated PI3K pathway, invasive adenocarcinoma, as well as resistant to ADT^15–17^. Cancer cell-intrinsic PI3K activation in the *Pten*-null model also promotes an immune suppressive microenvironment, including increased immune suppressive myeloid-derived suppressor cells (MDSC) and regulatory T cells (Treg), decreased dendritic cell maturation, as well as decreased T cell infiltration and activation^18–21^. These results suggest that inhibiting cancer cell-intrinsic immune suppressive activity induced by PI3K activation may be a prerequisite for promoting T cell infiltration and achieving anti-tumor immunity.

Targeting the PI3K pathway is not a simple task, especially to achieve synergy with ICT, as PI3K pathway plays significant roles on both sides of the aisle. As one of the most important oncogenic pathways, PI3K activation promotes cell proliferation, survival, migration, angiogenesis, metabolic reprograming as well as an immune suppressive environment^22–25^; on the other hand, PI3K is also a critical regulator for the functions of immune cells within the tumor microenvironment, and inhibition of PI3K activity in these immune cells may be detrimental for ICT^26–28^. To make things even more complicated, there are four isoforms in the class I PI3K family: PI3Kα/β are ubiquitously expressed and often abnormally activated in cancer cells by constitutively activated *PI3KCA* mutations or loss-of-function *PTEN* mutations, while PI3Kδ/γ are commonly restricted to leukocytes and essential for immune surveillance. Various immune cells within the tumor microenvironment are preferentially relying on different isoforms of the PI3K to promote or inhibit tumor development^24,29^.

We recently report that, BAY1082439, a selective PI3K inhibitor with equal potency against the PI3Kα/β/δ isoforms, is highly effective in inhibiting primary and CRPC in the *Pten*-null model^30^. However, the effects of BAY1082439 on alleviating cancer cell-intrinsic immunosuppressive activity and on various types of immune cells within the tumor microenvironment, particularly CD8^+^ T cells, have not been investigated. In this study, we report that by changing the dosing schedule from daily to intermittent, BAY1082439 can generate favorable anti-tumor immune response through alleviating cancer cell-intrinsic immunosuppressive activity, directly inhibiting Treg cells, promoting IFNα/γ pathway activation, CD8^+^ cell infiltration and clonal expansion. As a consequence, intermittent treatment of BAY1082439 paved the way for effective ICT therapy.

## Results

### *Pten*-null prostate cancers are poorly T cell infiltrated and resistant to anti-PD-1 immunotherapy

Most prostate cancers are immunogenically “cold” with low number of infiltrating T cells although ADT is known to promote T cell infiltration^12,19^. We confirmed this clinical observation on the *Pten*-null prostate cancer model. Immunohistochemistry (IHC) staining of prostate tissues from the *Pten*-null mice showed that CD8^+^ T cells were scarcely present in the intact mice but increased upon castration (Supplementary Fig. 1A). However, most of the T cells remained in the stroma area and could not penetrate into the tumor acini even after castration (Supplementary Fig. 1A).

We then sought to test if T cells-induced upon castration could lead to anti-tumor immunity in the presence of ICT. Castrated *Pten*-null mice were treated with either the anti-PD-1 or isotype control antibody for 4 weeks (Supplementary Fig. 1B). FACS analysis revealed that although tumor-associated CD8^+^ T cells and Treg cells were slightly increased upon anti-PD-1 treatment, the CD8^+^ /Treg ratio remained unchanged in comparison to the control antibody (Supplementary Fig. 1C). Hematoxylin and eosin (HE) and IHC assessments further confirmed that the numbers and localization of CD8^+^ and Granzyme b^+^ (GZMb^+^) cells after anti-PD-1 therapy remained unchanged (Supplementary Fig. 1D). Therefore, the *Pten*-null prostate cancer model mimics human prostate cancers with poor T cell infiltration and resistance to anti-PD-1 monotherapy. *Pten*-null prostate cancer model could serve as an in vivo model to investigate the molecular mechanism underlying the T cell exclusion phenotype-associated with human prostate cancers.

### BAY1082439 inhibits the cancer cell-intrinsic growth and immunosuppression and activates IFNα and IFNγ pathways

We have shown in our previous study that PTEN-loss in the prostatic epithelial cells promotes immunosuppressive microenvironment during prostate cancer initiation and progression but the underlying mechanisms are largely unknown^18^. We hypothesized that PTEN-loss or PI3K pathway activation in cancer cells may modulate anti-cancer immune response, which dampens the immune activities of cells within the tumor microenvironment.

**Fig. 1:**
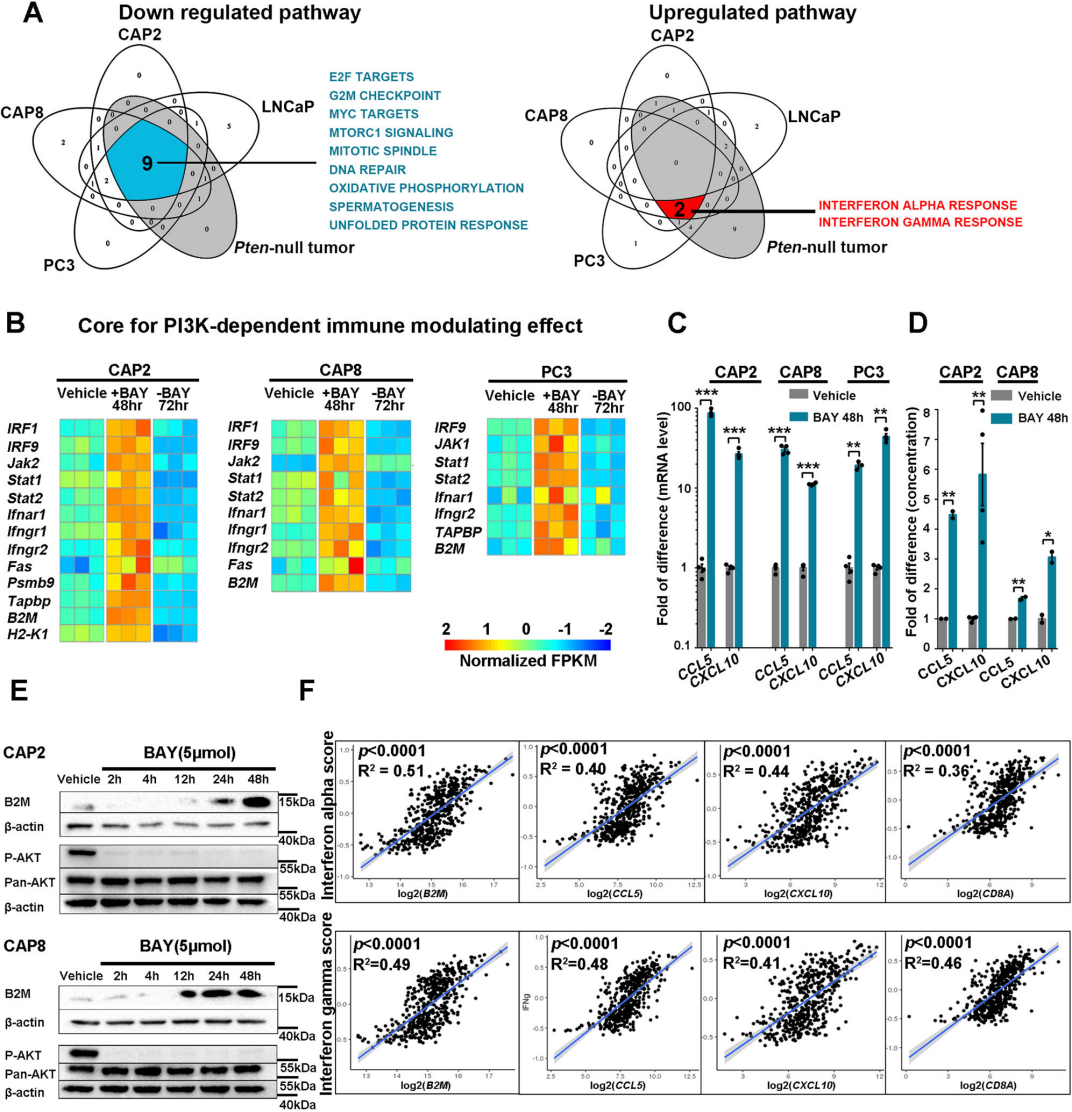
BAY1082439 inhibits the cancer cell-intrinsic immunosuppressive activity. **A** PTEN null CAP2, CAP8, PC3 and LNCaP prostate cancer cell lines and *Pten*-null prostate cancer in vivo model were treated with vehicle or 5μM BAY1082439 for 48 h (for cell lines), or 180 mg/kg bullet dose (for in vivo model). RNA-seq and GSEA analyses were performed and commonly down- or up-regulated pathways were presented with *p* < 0.05 as significant. Statistical test was performed by GSEA. **B** RNA-seq analysis identifies the core for PI3K-dependent immune modulating effect. RNAseq data from (**A**) and 72 h after BAY1082439 withdrawal were analyzed. The relative gene expression levels of indicated genes in each sample and cell line were determined and presented as heatmaps with *p* < 0.05 by two-sided T test (except in CAP8 cell line, *p* = 0.264 for *STAT1* and p = 0.067 for *Fas*). **C** RT-PCR analyses showed the relative expression levels of *CCL5/CXCL10* chemokines. **D** ELISA measurement for the relative CCL5/CXCL10 secretion levels. PTEN null CAP2 and CAP8 cells were treated with BAY1082439 or vehicle for 48 h. Culture supernatant was collected and analyzed as suggested by manufactural recommendation. **E** Western blot analyses for P-AKT, Pan-AKT and B2M levels. PTEN null CAP2 and CAP8 cells were treated with BAY1082439 or vehicle for indicated time periods, and cell lysates were analyzed by Western blot using indicated antibodies. The experiment was repeated 3 independent times with similar results. **F** The positive correlations between IFNα/γ activity scores and CCL5/CXCL10/B2M/CD8A gene expression levels in human prostate cancer samples (499 patients). Linear regression was used, error bands represent 95% confidence intervals. Statistical test done by two-sided T test. **C**-**D**: each experiment was repeated 3 times and mean ± SEM were presented in **C**-**D** with * *p* < 0.05, ** *p* < 0.01, *** *p* < 0.001 by two-sided *T* test. Source data and exact *p* values are provided in the Source Data file.

We treated *Pten*-null prostate cancer-derived cell lines CAP2 and CAP8^16^ and *PTEN*-null human prostate cancer cell line PC3 and LNCaP with BAY1082439 to investigate the effects of BAY1082439 on cancer cell-intrinsic properties. As expected for PI3K pathway inhibition, global gene expression analysis demonstrated that BAY1082439 treatment led to downregulation of mTOR signaling and cell proliferation-related pathways as well as Ki67 expressions in all 4 prostate cancer cell lines (Fig. 1A; Supplementary Fig. 2A; Supplementary Data 1). Interestingly, the interferon α and γ (IFNα and IFNγ) response pathways were the 2 commonly upregulated pathways found in BAY1082439 treated CAP2/CAP8/PC3 lines but not in LNCaP line, consistent with previous report that LNCaP does not respond to IFNα and IFNγ pathway activations^31^ (Fig. 1A; Supplementary Fig. 2B). Importantly, the effects of BAY1082439 on inhibition of cell proliferation and PI3K, IFNα and IFNγ pathways could be observed 24–48 h after a bullet dose on the *Pten*-null prostate cancer model in vivo (Fig. 1A; Supplementary Fig. 2C and E; Supplementary Data 1) without influence AR expression levels (Supplementary Fig. 2D).

Quantitative RT-PCR analysis further revealed that IFN-regulated transcription factors, such as *IRF1*, *IRF2*, *IRF3*, *IRF7* and *IRF9*, were upregulated in CAP2/CAP8/PC3 cell lines, but not in LNCaP line, in a time-dependent manner (Supplementary Fig. 2B). Besides the JAK-STAT pathway, the immune modulating effects of BAY1082439 appeared to be PI3K activation-dependent as inducing *PTEN* re-expression in the *PTEN*-null PC3 cells could significantly diminish the effects of BAY1082439 (Supplementary Fig. 2F).

To further determine the cancer cell-intrinsic and PI3K activation-dependent immune modulating effects, we analyzed the RNAseq date from CAP2/CAP8/PC3 cell lines with two criteria: gene expressions were upregulated upon 48 h BAY1082439 treatment and downregulated 72 h after BAY1082439 withdrawal. We found that genes within the Interferon-JAK-STAT axis and antigen presentation fitted these criteria well and were the core components (Fig. 1B). Importantly, many genes within this core were known to cause resistance to anti-PD-1 therapy when deleted^32^, and substantially overlapped with those recently identified with cancer-intrinsic evasion of killing by T cell function^33^. As a result of IFNα and IFNγ pathway activation, the expressions of *CCL5* and *CXCL10*, chemokines known to have pleiotropic effects on monocytes, NK and T cell migration and activation of T cell proliferations^34^, were also upregulated (Fig. 1C). Finally, ELISA and Western bolt analysis confirmed that BAY1082439 treatment indeed led to increased CCL5 and CXCL10 secretion and B2M expression from CAP2 and CAP8 cells (Fig. 1D–E).

The strong positive correlations between IFNα and IFNγ pathway activities and *CCL5*, *CXCL10, B2M* and *CD8A* gene expressions could also be observed in human prostate, lung and melanoma cancers samples (Fig. 1F; Supplementary Fig. 2G-H). Together, these results suggest that BAY1082439 treatment may convert the PTEN-loss induced cancer cell-intrinsic immuno-suppression to immuno-stimulation by upregulating IFNα and IFNγ pathways, increasing cancer cell antigen-presentation, and releasing T cell attractive chemokines.

### Intermittent but not daily BAY1082439 treatment turns *Pten*-null prostate tumors to T cell-inflamed

**Fig. 2:**
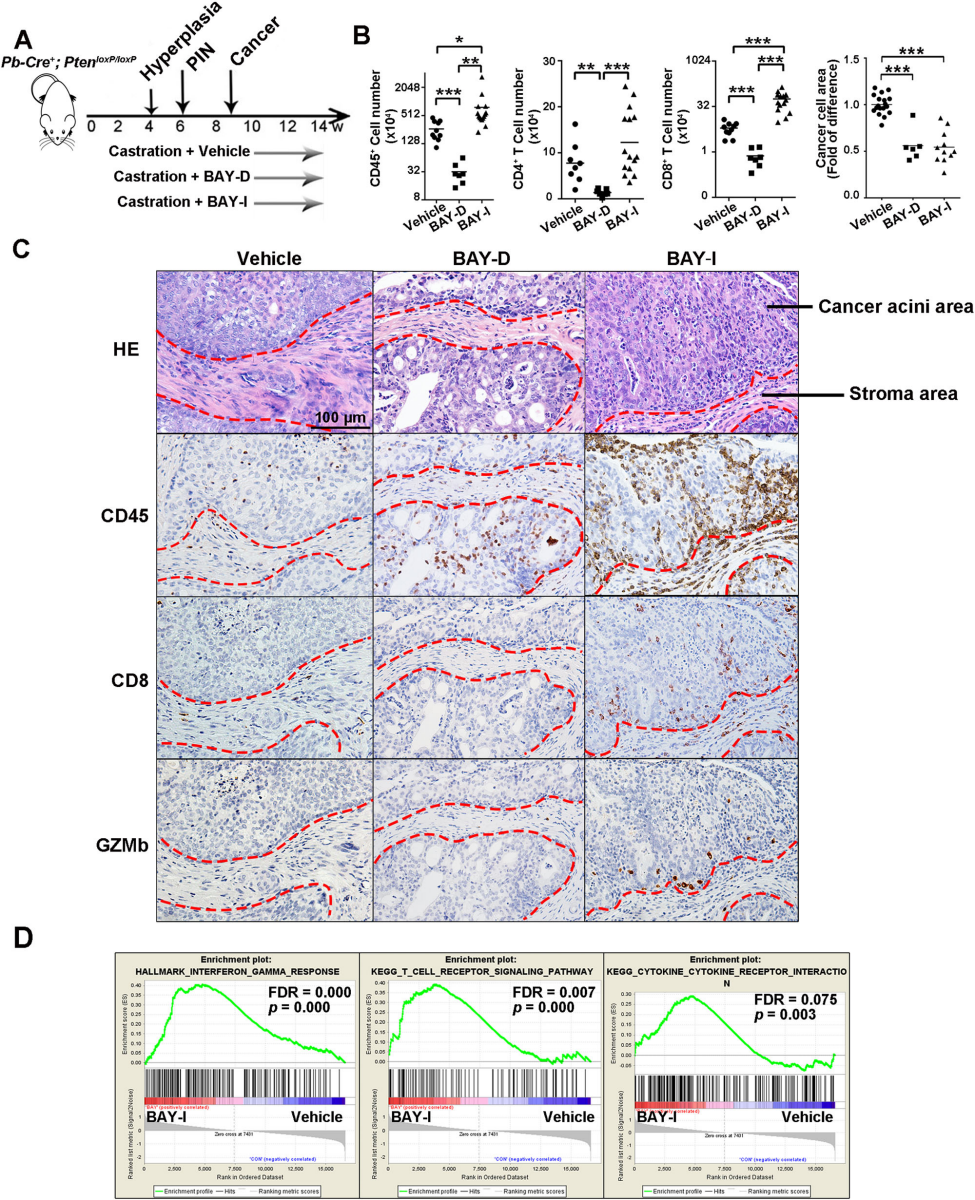
Intermittent but not daily BAY1082439 treatment turns *Pten*-null prostate tumors to T cell-inflamed. **A** A schematic illustration of treatment schedules. **B** FACS analyses for tumor-associated immune cells. Castrated *Pten*-null mice were treated with vehicle (*n* = 18), BAY-D (*n* = 7) or BAY-I (*n* = 11) for 4 weeks. Tumor tissues were dissociated and weighted, the numbers of tumor-associated CD45^+^, CD4^+^ and CD8^+^ T cells were measured by FACS analysis. Prostates were fixed and stained with HE, and cancer cell areas in anterior lobes were measured and fold of differences between vehicle and treatment groups were presented. Data were presented as dot plots with mean as the central lines; *p < 0.05; **p < 0.01, ***p < 0.001 by two-sided T-test. **C** H&E and Immunohistochemistry analyses for immune cell infiltration. Consecutive sections were stained with H&E or antibodies against CD45, CD8 and GZMb. Dashed red lines: the boundaries between cancer acini and stroma areas. The same staining was performed with 6 mice in vehicle and BAY-I cohort and 7 mice in BAY-D cohort and similar results were observed. **D** RNA-seq and GSEA for BAY-I responses. RNAs were extracted from BAY-I treated tumor tissues for RNA-seq analysis. GSEA analysis showed enriched IFN-γ, T cell reporter and cytokine-cytokine receptor interaction signaling pathways in BAY-I treated cohort. Statistical test was performed by GSEA. Source data and exact *p* values are provided in the Source Data file.

The potent effect of BAY1082439 in inhibiting cancer cell-intrinsic immunosuppressive activity prompted us to test whether BAY1082439 treatment could turn *Pten*-null prostate cancer to T cell-inflamed and promote T cell-mediated anti-tumor immunity in vivo. Unfortunately, daily 75 mg/kg BAY1082439 treatment led to significantly decreased tumor- and spleen-associated CD45^+^, CD8^+^ T and CD4^+^ T cell numbers (Fig. 2A, B; Supplementary Fig. 3A), which could be detrimental for T cell-mediated anti-tumor immunity even if cancer cell-intrinsic immunosuppression could be alleviated.

We therefore searched for alternative dosing schedule, including intermittent and weekly treatment, to effectively target cancer cells while reducing immune toxicity effect^35^. We first compared the target effects among 75 mg/Kg daily, 2-day on/5-day off 200 mg/Kg intermittent and 450 mg/Kg weekly schedules, respectively, for a week on GXA3027 PDX model. Tumor tissues were harvest at 2, 8, 24, 48, 96 and 168 hr post the 1^st^ dose and P-AKT levels were measured (Supplementary Fig. 3B). With 75 mg/kg daily schedule, inhibition of P-AKT was observed at 2 and 8 h, but diminished before the next dosage, while with 200 mg/Kg 2On/5Off schedule, substantial inhibition of P-AKT ( > 70%) was observed within the first 48 h, reduced inhibition started from 96 h (day 4) and diminished on day 7. With 450 mg/Kg weekly schedule, complete P-AKT inhibition (>80%) was observed in the first 24 h, weaker inhibition at 96 h compared to 2On/5Off schedule and completely diminished P-AKT inhibition on day 7. Therefore, 2On/5Off intermittent schedule had a much longer coverage for anti-PI3K effect (Supplementary Fig. 3B).

We then tested the long-term effects of 75 mg/Kg daily (BAY-D) and 180 mg/Kg intermittent (BAY-I) dosing schedules on 10 week-old castrated *Pten*-null prostate cancer mice when prostate developed high grade PIN/adenocarcinoma phenotypes^15,36^ (Fig. 2A). BAY-D or BAY-I treatment did not cause significant and persistent animal body weight reduction or reach humane endpoint^37^ (Supplementary Fig. 3C).

Despite of a significant reduction in overall dosage in the BAY-I cohort (1440 mg/Kg total dose for BAY-I vs. 2100 mg/kg total dose for BAY-D), both BAY-D and BAY-I showed equal potency in decreasing cancer cell burden after 4 weeks of treatment (Fig. 2A, B right panel) without significant change the pathological features associated with *Pten*-null CRPC, including nuclear atypia, loss or partially loss of CK5 basal cells and SMA staining and immune cell infiltration (Fig. 2C and Supplementary Figs. 3D and  4A).

We and others have shown the important roles of MDSC in establishing immunosuppressive microenvironment in the *Pten* null model^18,20,21,28^. When we conducted quantitative FACS analysis on prostate cancer-associated immune cells, we found that B220^+^ B cells were almost completely depleted in BAY-D and BAY-I treated prostates, consistent with our previous study^30^, while CD11b^+^Gr-1^+^ MDSC did not change significantly (Supplementary Fig. 4B). In contrast to BAY-D, BAY-I treatment led to significantly increased prostate cancer-associated CD45^+^ and CD8^+^ T cell numbers, and CD8^+^ T cell percentage in CD45^+^ cell without altering spleen associated CD45^+^ cell, CD4^+^ T cell and CD8^+^ T cell numbers (Fig. 2B, C; Supplementary Fig. 3A), so we decided to focus our current study on the effects of BAY-I on T cells.

The effects of BAY-I treatment on CD8^+^ T cells appeared to be tumor-specific, as CD8^+^ T cell numbers were not changed in other organs, such as the spleen, lung and liver, in the same animals (Supplementary Fig. 3A and Fig. 4C). Importantly, BAY-I, but not BAY-D, treatment could break “the immuno-protective barrier” and promote CD8^+^ and GZMb^+^ cells penetration into cancer acini (Fig. 2C). The effects of BAY-I treatment on T cell activation could also be observed in intact *Pten*-null prostates (Supplementary Fig. 4D), suggesting that BAY-I could be used in both primary and CRPC settings. In addition, in *Pb-Cre*^*+*^*;Pten*^*loxP/loxP*^*;K-ras*^*G12D/W*^ model^38^, which represents more aggressive type of prostate cancer^36,38^, BAY-I showed similar CD8^+^ T cell promoting effect (Supplementary Fig. 5A-C).

Bulk tumor tissue RNA-seq analysis revealed that BAY-I treatment led to significantly increased IFNγ, T-cell receptor and cytokine-cytokine receptor signaling pathways (Fig. 2D). RT-PCR analysis further confirmed that the *Ifn*γ expression was increased in BAY-I treatment cohort (Fig. 6E). Thus, intermittent but not daily BAY1082439 treatment could convert the immunosuppressive tumor microenvironment-associated with the *Pten*-null prostate cancer to a T cell-inflamed one.

### Tregs are hypersensitive to BAY1082439 and intermittent BAY1082439 treatment leads to increased tumor-associated CD8^+^/Treg ratios

**Fig. 3:**
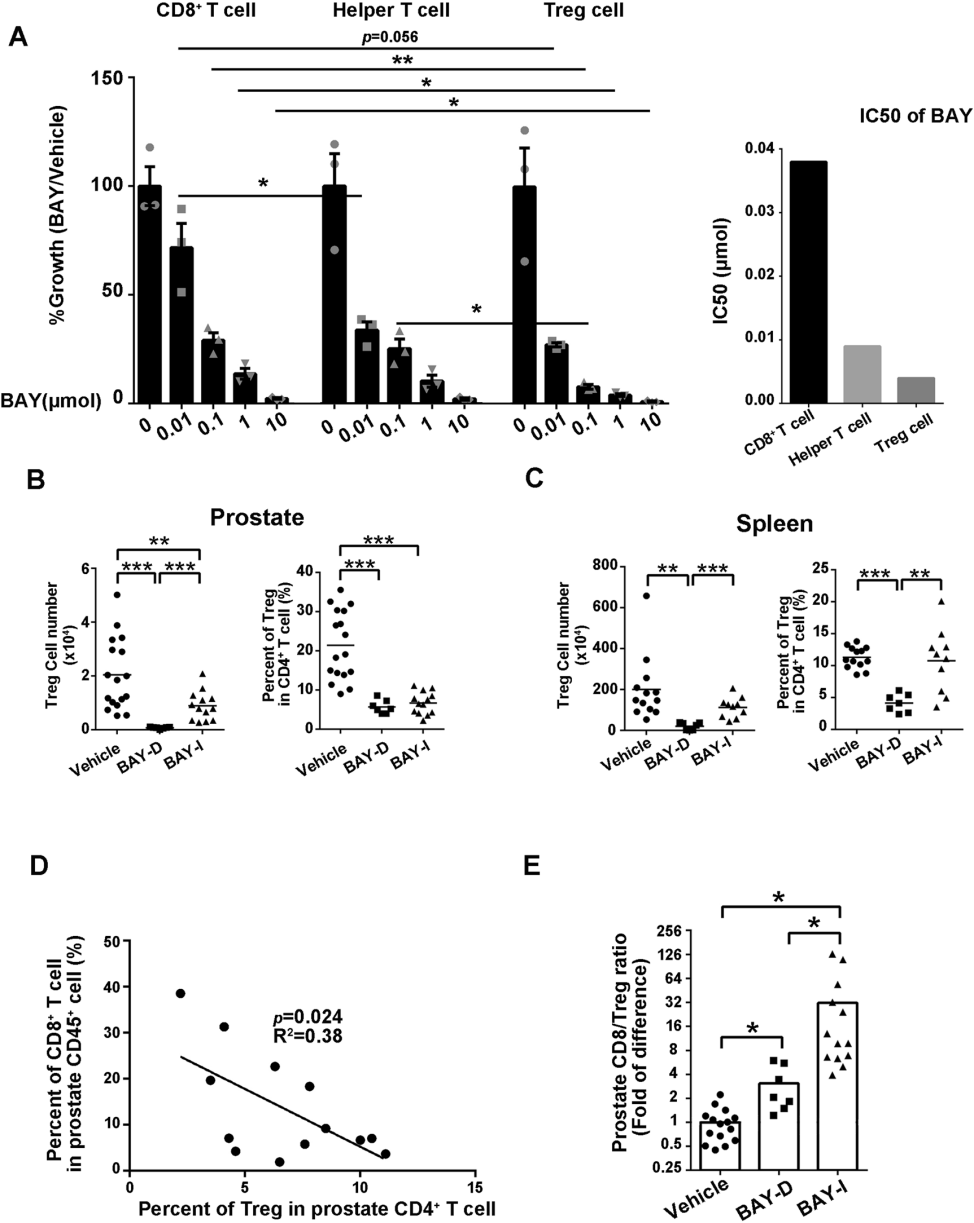
Tregs are hypersensitive to BAY1082439. **A** The differential inhibitory effects of BAY1082439 on T cells. Primary CD8^+^, helper T and Treg cells from spleen of WT mice were FACS sorted, and cultured with different concentrations of BAY1082439. This experiment was repeated 3 times the percentages of cell growth as compared to vehicle controls were presented. Data were presented as mean ± SEM; *p < 0.05, **p < 0.01, ***p < 0.001 by two-sided T-test. IC50s were calculated by GraphPad Prism 6 software. **B**, **C**. Prostate- and spleen-associated Treg cell numbers and the percentages of Treg in CD4^+^ T cell in vehicle (*n* = 17), BAY-D (*n* = 7) and BAY-I (*n* = 13) treated cohorts were analyzed by FACS. Data were presented as dot plots with mean as the central lines; **p < 0.01, ***p < 0.001 by two-sided T-test. **D** The negative correlation between the percentages of CD8^+^ in CD45^+^ and Treg in CD4^+^ cells by Pearson correlation coefficient. **E** The CD8^+^/Treg ratios in vehicle, BAY-D and BAY-I treated cohorts analyzed by FACS. Data were presented as dot plots with mean as the central lines; *p < 0.05 by two-sided T-test. Source data and exact *p* values are provided in the Source Data file.

We next tested the effects of BAY1082439 on different subtypes of T cells as we observed significant increased CD8^+^ T cells and decreased Tregs in cancer associated immune cells in vivo in BAY-I treated cohort (Supplementary Figure 4B and Supplementary Fig. 4D). By treating freshly isolated CD8^+^, CD4^+^/CD25^-^ helper and CD4^+^/CD25^+^/CD127^low/−^ Treg cells with different concentrations of BAY1082439, we found that Tregs were most sensitive to BAY1082439, followed by T helper, while CD8^+^ T cells were the least sensitive to BAY1082439; and the calculated IC50 for CD8^+^ T cells was approximately 10 times higher than that of Treg (Fig. 3A, right panel). Similar tests showed that BAY108 was more potent than PI3Kδi CAL-101 and superior than PI3Kβi TGA-221 and AKTi Ipatasertib in inhibiting T and B cells, consistent with previous studies (Supplementary Table 3)^23,39–41^.

We then quantified tumor-associated Tregs in the *Pten*-null model in vivo. Both BAY-D and BAY-I treatments led to a significantly decreased total tumor-associated Treg numbers and the percentage of Treg in CD4^+^ T cells (Fig. 3B). However, BAY-D, but not BAY-I, treatment also led to decreased spleen-associated Treg numbers and the percentage of Treg in CD4^+^ T cells in the same animals (Fig. 3C), suggesting that intermittent treatment could minimize aberrant immune activation in non-cancerous organs.

The percentages of CD8^+^ in CD45^+^ were negatively correlated with the percentages of Treg in CD4^+^ T cells in vivo in the BAY-I treatment cohort (Fig. 3D). Importantly, BAY-I treatment could dramatically increase the intra-tumoral CD8^+^/Treg ratio by 32-fold, as compared to only 3-fold increase in the daily treatment group (Fig. 3E). Therefore, BAY-I treatment could alleviate both cancer cell-associated and Treg-mediated immuno-suppressions, and allow CD8^+^ T cell expansion and activation.

### Intermittent BAY1082439 treatment induces intratumoral CD8^+^ T cell clonal expansion

**Fig. 4:**
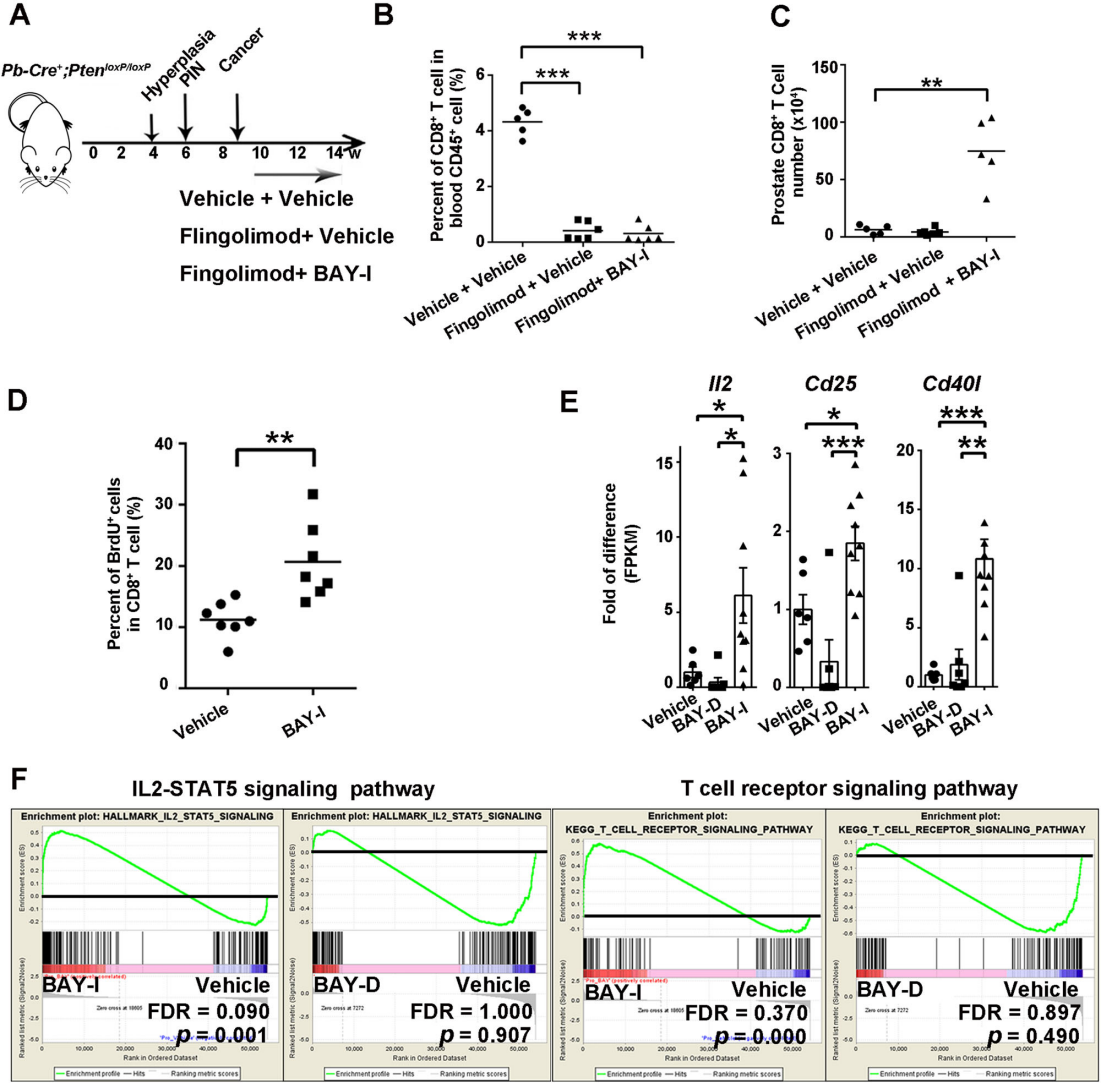
Intermittent BAY1082439 treatment induces tumor-associated CD8^+^ T cell clonal expansion and activation of the IL-2 and TCR signing pathways. **A**–**C**. A schematic illustration of treatment procedures and the percentages of CD8^+^ in CD45^+^ in peripheral blood and the prostates. *Pten*-null mice were treated with vehicle (*n* = 6), S1PR modulator Fingolimod alone (*n* = 5) or in combination with BAY-I (*n* = 6) for 4 weeks. CD45^+^ and CD8^+^ T cells from peripheral blood or the prostates were sorted and presented as the percentage of CD8^+^ in CD45^+^ cells. **D** BAY-I treatment induced CD8^+^ T cell proliferation. *Pten*-null mice was treated with vehicle (*n* = 7) or BAY-I (*n* = 7) for 2 weeks, then BrdU labeled (10 mg single dose) for 24 h before analyzing the percentages of BrdU^+^ cells in CD8^+^ T cell by FACS analysis. **E** BAY-I treatment induced CD8^+^ T cell activation. Castrated *Pten*-null mice were treated with vehicle (*n* = 6), BAY-D (*n* = 7) or BAY-I (*n* = 9) for 4 weeks, and tumor-associated CD8^+^ T cells were sorted then analyzed by RNA-seq. The relative expression levels of *Il2*, *Cd25* and *Cd40l* were presented. **F** GSEA analysis of IL2 and TCR single pathway between Vehicle and BAY-I/BAY-D group. Statistical test was performed by GSEA. Data were presented as dot plots with mean as the central lines (for **A**, **B**, **C** and **D**) or as mean ± SEM (for **E**); *p < 0.05, **p < 0.01, ***p < 0.001 by two-sided T-test. Source data and exact *p* values are provided in the Source Data file.

To study whether the increased CD8^+^ T cells seen in the BAY-I treated prostates were derived from local expansion or recruited from peripheral blood, we treated the *Pten*-null mice with Fingolimod, a S1P receptor modulator that could block lymphocyte egress from hematopoietic organs and lymph nodes^42^ (Fig. 4A). Fingolimod treatment effectively depleted T cells in the peripheral blood, but had no effect on BAY-I induced CD8^+^ T cell expansion within the prostates (Fig. 4B, C), suggesting that increased CD8^+^ T cells after BAY-I treatment were mainly from intra-tumoral expansion rather than peripheral recruitment. This notion was further supported by BrdU-pulse labeling experiment, as BAY-I treatment doubled the percentage of prostate-associated CD8^+^ cells in cell cycle (Fig. 4D).

We next conducted RNA-seq analysis on CD8^+^ T cells isolated from the prostates and found significantly increased *Il2* as well as T cell activation markers *Cd40l* and *Cd25* expressions in BAY-I treated, but not in BAY-D treated cohort (Fig. 4E; Supplementary Data 1). GSEA pathway analysis revealed enrichment for IL2-STAT5 and T cell receptor signaling pathway in BAY-I but not in BAY-D treated CD8^+^ T cells (Fig. 4F). TCR analysis^43^ also revealed a significant decrease in clonotype diversity of tumor-associated, but not in spleen-associated, CD8^+^ T cells upon BAY-I treatment (Fig. 7F and Supplementary Figure 6). Given BAY1082439 treatment can significantly increase the expression of *B2M* in *PTEN*-null prostate cancer cells (Fig. 1B and E), this result indicates that BAY-I treatment may induce proliferation and activation of tumor antigen-specific T cells.

### Intermittent BAY1082439 treatment-induced anti-tumor immunity is CD8^+^ T cell-dependent

**Fig. 5:**
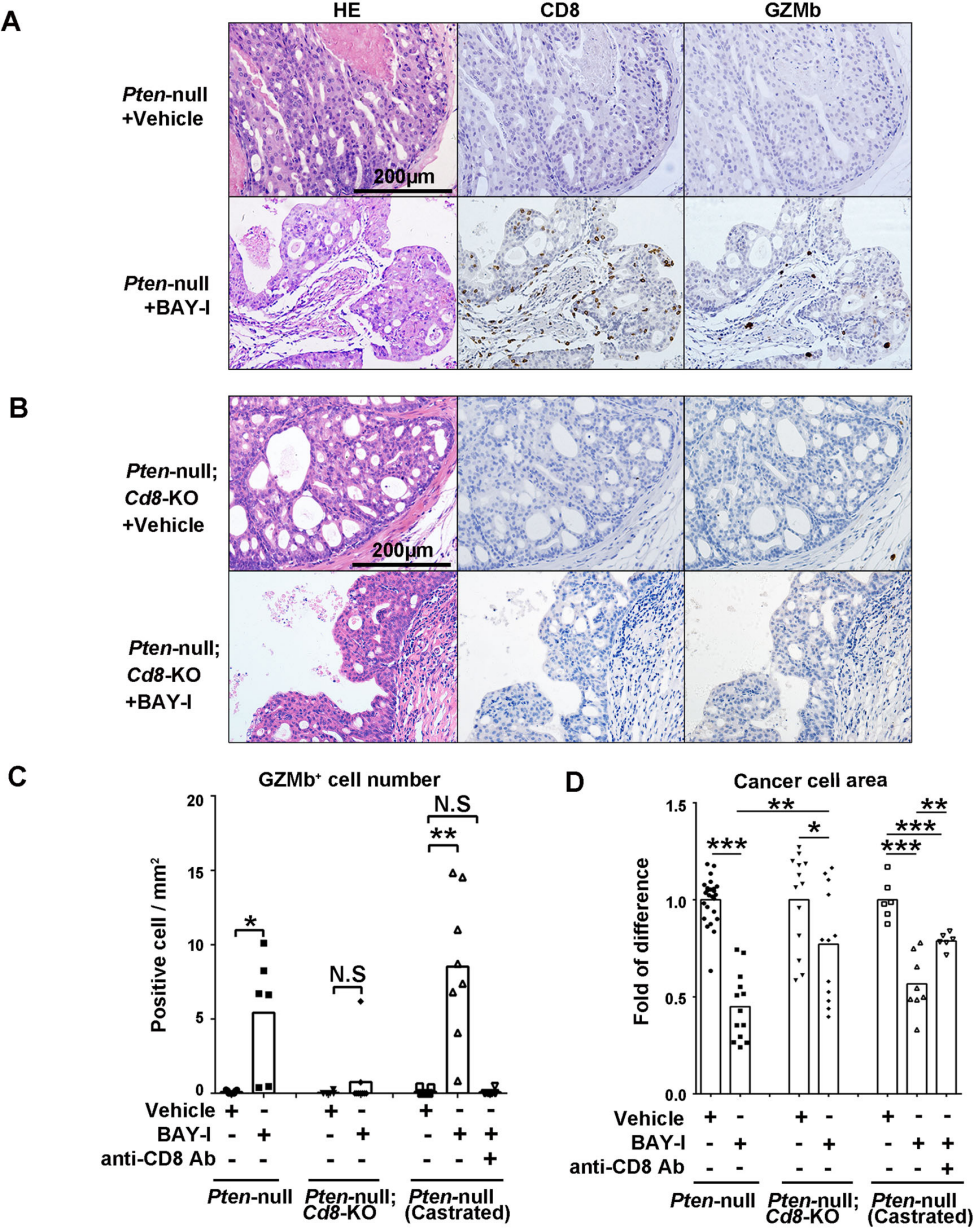
Intermittent BAY1082439 treatment-induced anti-tumor immunity is CD8^+^ T cell-dependent. **A**, **B**. BAY-I induced anti-tumor immunity was diminished in *Pten*-null;*Cd8*-KO mice. 10 weeks old *Pten-null* and *Pten*-null;*Cd8*-KO mice were treated with vehicle (*n* = 25 for *Pten*-null mice, *n* = 13 for *Pten*-null;*Cd8*-KO mice) or BAY-I (*n* = 13 for *Pten*-null mice, *n* = 12 for *Pten*-null;*Cd8*-KO mice) for 4 weeks. Tumor tissues were harvested and fixed, and consecutive sections were stained for H&E and antibodies against CD8 and GZMb. **C**, **D**. BAY-I treatment efficacies in prostate from *Pten*-null (*n* = 25 in vehicle cohort, *n* = 13 in BAY-I cohort), *Pten*-null;*Cd8*-KO (*n* = 13 in vehicle cohort, *n* = 12 in BAY-I cohort), castrated *Pten*-null with (*n* = 6) or without anti-CD8 antibody (*n* = 6 in vehicle cohort, *n* = 8 in BAY-I cohort) were determined by counting GZMb^+^ cells density in cancer areas (**C**), and calculating anterior lobe’s cancer cell area in H&E stained slides (**D**) for each treatment cohorts. Data were presented as dot plots with mean as the central lines; N.S *p* > 0.05, *p < 0.05, **p < 0.01, ***p < 0.001 by two-sided T-test. Source data and exact *p* values are provided in the Source Data file.

To demonstrate that the BAY-I treatment-induced anti-tumor immunity is CD8^+^ T cell-dependent, we crossed the *Pten*-null mice with CD8-KO mice^44^ and generated *Pten*-null;*Cd8-*KO double knockout mice. *Pten*-null;*Cd8-*KO mice developed prostate cancer with similar characteristics as the *Pten*-null mice (Fig. 5A and Supplementary Fig. 7A), indicating that CD8^+^ T cells play little role during tumorigenesis in the *Pten*-null mice. Although BAY-I treatment had similar effects on tumor-associated CD4^+^ T cell and Treg cells in the *Pten*-null;*Cd8-*KO mice (Supplementary Fig. 7B), the increased intratumoral CD8^+^ and GZMb^+^ cells seen in the *Pten*-null mice upon BAY-I treatment was nearly completely abolished in *Pten*-null;*Cd8-*KO mice (Fig. 5C). We next investigated BAY1082439 treatment efficacy in the presence or absence of CD8^+^ cells by quantifying cancer cell areas in the anterior lobes of the prostates. Comparing to 55% reduction in the *Pten*-null prostate, BAY-I treatment of the *Pten*-null;*Cd8-*KO mice only had 23% reduction of cancer cell areas (Fig. 5D). We also treated castrated *Pten*-null mice with a CD8 depletion antibody. Similar to that of genetic deletion, an antibody-mediated depletion of CD8^+^ T cell completely abolished the intratumoral GZMb+ cells and decreased cancer cell reduction effect of BAY-I treatment (Fig. 5C, D and Supplementary Fig. 7C). Collectively, these results suggest that BAY-I treatment not only directly inhibits *Pten*-null prostate cancer cell growth, but also triggers CD8^+^ T cell-dependent anti-tumor immunity and cancer cell killing effects.

### Intermittent BAY1082439 treatment induces prolonged T cell-inflamed phenotype even after drug withdrawal

**Fig. 6:**
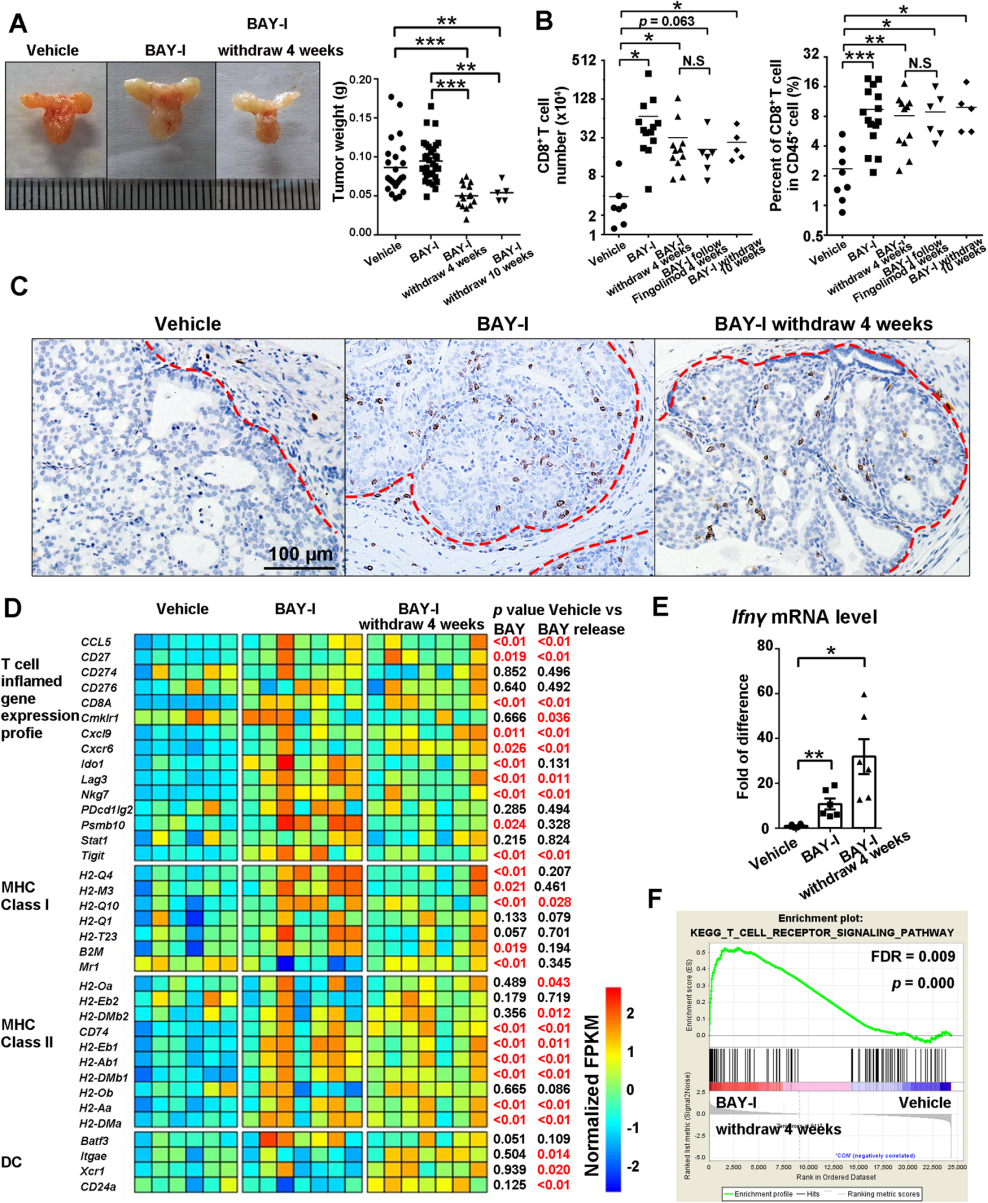
Intermittent BAY1082439 treatment induces prolonged T cell-inflamed phenotype even after drug withdrawal. **A**-**B** BAY-I treatment induced prolonged T cell-inflamed phenotype after drug withdrawal. Castrated *Pten*-null mice were treated with vehicle (*n* = 24), BAY-I (*n* = 32), or BAY-I then drug withdrawal for 4 (*n* = 15) weeks, BAY-I then drug withdrawal then S1PR modulator Fingolimod for 4 weeks (*n* = 6) or BAY-I then drug withdrawal for 10 weeks (*n* = 5) before the analyses. Prostate tumor sizes and weight were presented in (**A**), and prostate tumor-associated CD8^+^ T cell number and the percentages of CD8^+^ in CD45^+^ cells were determined by FACS analysis (**B**). **C** IHC analysis showed that CD8^+^ T cells remained in the cancer acini 4 weeks after drug withdrawal. Red dash line marks the boundary between cancer acini and stroma areas. The same staining was performed with 6 mice in vehicle and BAY-I cohort and 8 mice in BAY-I withdraw cohort and similar results were observed. **D** RNA-seq analysis shows T cell inflamed phenotype after drug withdrawal. RNAs were extracted from tumor tissues in (**A**, **B**), and RNA-seq analyses were performed. The relative expression levels of indicated genes in each sample were determined and the statistical analysis was performed based on the average of expression levels of each cohort. *n* = 6, 7 and 7 for vehicle, BAY-I and BAY-I withdraw 4 weeks group. *p* was calculated by two-sided T-test. **E** The relative expression levels of the *Ifn-γ* gene were determined by RT-PCR analysis. *n* = 6 at each treatment cohort. **F** The enrichment of T cell receptor pathway was determined by GSEA based on RNA-seq data in **E**. Statistical test was performed by GSEA. **A** and **B**, data were presented as dot plots with mean as the central lines; **E** Data were presented as mean ± SEM; N.S *p* > 0.05, *p < 0.05, **p < 0.01, ***p < 0.001 by two-sided T-test. Source data and exact *p* values are provided in the Source Data file.

We next tested whether the BAY1082439 induced T cell-inflamed phenotype persists without continuous drug administration for subsequent combination of ICT. Castrated *Pten*-null mice were treated with vehicle or BAY-I for 4 weeks, then the treatment was stopped for 4 or 10 weeks before the analyses. Interestingly, tumor size and weight were decreased significantly in both 4- and 10-week drug withdrawal groups, as compared to the vehicle or BAY-I group after the last dose (Fig. 6A). FACS analysis showed that the increased tumor-associated CD8^+^ T cell numbers and the percentage of CD8^+^ in CD45^+^ were well maintained 4- and 10-weeks after drug withdrawal, and were not affected by S1PR modulator Fingolimod (Fig. 6B). Importantly, intra-acini CD8^+^ T cell infiltration could be clearly visualized in the prostate tissues 4 and 10-weeks following drug withdrawal (Fig. 6C and data not shown). These results indicate that the BAY-I induced T cell-inflamed phenotype can persist for at least 10 weeks after drug withdrawal, and is likely maintained by intratumoral clonal expansion of tumor specific CD8^+^ T cells, rather than recruitment from the circulation.

RNA-seq analysis of the bulk tumor tissues revealed that BAY-I treatment could increase the T cell-inflamed gene expression profile^12^ and the expressions of MHC class I and II molecules, which were well maintained in 4-week drug withdrawal group (Fig. 6D; Supplementary Data 1). Importantly, the dendritic cell (DC)-associated genes were significantly upregulated in the drug withdrawal group (Fig. 6D). IFNγ gene expression and TCR pathway enrichment were not only maintained but further increased in the drug withdrawal group (Fig. 6E, F). Together, these results demonstrated that BAY-I treatment can prime the tumor and generate a persistent T cell inflammatory environment even in the absence of continued drug administration.

### Intratumoral tertiary lymphoid structures and CD8^+^ memory phenotype-associated with intermittent BAY1082439 treatment

**Fig. 7:**
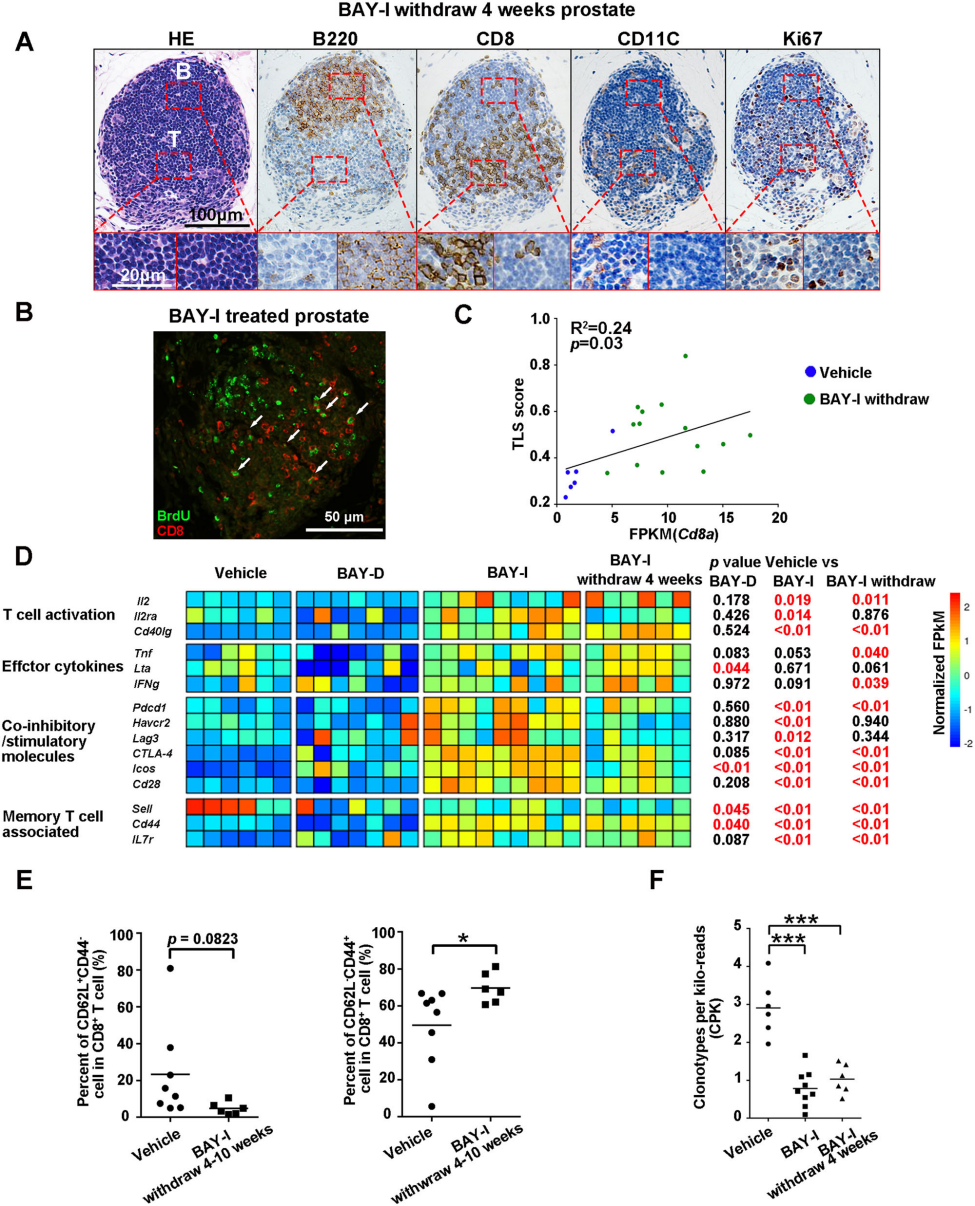
Intratumoral tertiary lymphoid structures and BAY1082439-induced CD8^+^ memory phenotype. **A** H&E and IHC analyses showing a representative tertiary lymphoid structure in BAY-I treated prostates. Castrated *Pten*-null mice were treated with 4 cycles of BAY-I then drug was withdrawal for 4–10 weeks. Tumor tissues were fixed, and tertiary lymphoid structures were determined by staining consecutive sections with H&E, and CD8 \B220\CD11C\Ki67 antibodies. The same staining were performed with 3 mice and similar results were observed. **B** CD8^+^ cells within the tertiary lymphoid structures are highly proliferative. BAY-I treated Animals were pulse-labeled by a bullet dose of BrdU before the analysis. Tumor tissues were fixed and CD8^+^BrdU^+^ double positive cells were visualized by co- Immunofluorescence staining with anti-BrdU and CD8 antibodies. The same staining were performed with 3 mice and similar results were observed. **C** TLS score is increased in BAY-I treated cohort and positively correlates with *Cd8a* expression. Castrated *Pten*-null mice were treated with 4 cycles of vehicle, BAY-I then withdrawal for 4 weeks, or treated with anti-PD-1 antibody as indicated in Fig. 8. TLS score was calculated from tumor RNAseq dataset. Correlation between TLS score and *Cd8a* expression was calculated. Statistic was performed by Pearson correlation coefficient. **D** BAY-I treated and BAY-I treated then drug withdrew prostate-associated CD8^+^ T cells remained activated. Castrated *Pten*-null mice were treated with vehicle (*n* = 6), BAY-D (*n* = 7) and BAY-I for 4 weeks (*n* = 9), and BAY-I for 4 weeks then drug withdrew for 4 weeks (*n* = 6). Tumor-associated CD8^+^ T cell were sorted by FACS. The relative expression levels of genes associated with T cell activation, effector cytokines, co-inhibitory/stimulatory molecules and T memory were presented based on RNA-seq data. *p* was calculated by two-sided T-test. **E** Cell surface expression levels of CD44 and CD62L molecules on tumor-associated CD8^+^ T cell in vehicle (*n* = 8) or BAY-I withdraw cohort (*n* = 6) mice were determined by FACS. *p = 0.0386 by two-sided T-test. **F** Vehicle (*n* = 6), BAY-I (*n* = 9) treated and BAY-I treated then drug withdrew (*n* = 6) prostate-associated CD8^+^ T cells remained clonal selected. TCR clonotype diversities were calculated and presented as dot plots with mean as the central lines; ***p < 0.001 by two-sided T-test. Source data and exact *p* values are provided in the Source Data file.

Effective priming of tumor antigen-specific T cells requires secondary lymphoid organs such as lymph nodes^6^. Since Fingolimod, a S1PR modulator that could block lymphocyte egress from hematopoietic organs and lymph nodes^42^, did not inhibit intratumoral CD8^+^ T cell activation/expansion induced by BAY-I treatment, we sought alternative mechanisms to explain our findings. In *Pten*-null prostate tumor tissues, we found tertiary lymphoid structures (TLS) with clear B and T cell zones, resembling germinal center morphology^45^ (Fig. 7A). CD11C^+^ dendritic cell and Ki67^+^ lymphocytes were also enriched in the T cell zone of TLS, indicating potential cross-presenting of tumor antigens (Fig. 7A). When pulse labeled with BrdU, substantial BrdU^+^CD8^+^ double positive cells were found in these intratumoral TLS structures in BAY-I treated group (Fig. 7B). We next calculated “TLS score^46^” using RNAseq data from vehicle and BAY-I withdrawal prostate samples, and found that BAY-I treated prostate had higher “TLS score” and was positively correlated with *Cd8a* expression (Fig. 7C). Together, these results indicate that the TLS structures may provide the necessary niche for intratumoral CD8^+^ T cell priming and clonal expansion after BAY-I treatment.

The TLS may also account for persistent T cell-inflamed phenotype after drug withdrawal. Comparing RNA-seq data of CD8^+^ T cells revealed increased expression levels of genes associated with T cell activation, effector cytokines, co-inhibitory/stimulatory molecules in the BAY-I and drug withdrawal groups, but not BAY-D treatment group (Fig. 7D; Supplementary Data 1). The RNA-seq data also revealed a decreased CD62L but increased CD44 and IL7R expression, indicating a shift to memory T cell phenotype. Indeed, FACS analysis demonstrated decreased naïve T cell (CD62L^+^CD44^-^) and increased effector-memory T cell (CD62L^-^CD44^+^) within tumor-associated CD8^+^ T cells in BAY-I treated withdrawal cohort as compared to vehicle cohort (Fig. 7E). Similar to BAY-I group, CD8^+^ T cells isolated from the drug withdrawal group also had decreased clonotype diversity (Fig. 7F). Taken together, these results indicate that BAY-I treatment can effectively trigger long-term inhibition of tumor growth even after drug withdrawal by intratumorally activating CD8^+^ T cells, leading to clonal expansion and effector memory phenotype, most likely via TLS.

### Intermittent BAY1082439 treatment paves the way for anti-PD-1 therapy

**Fig. 8:**
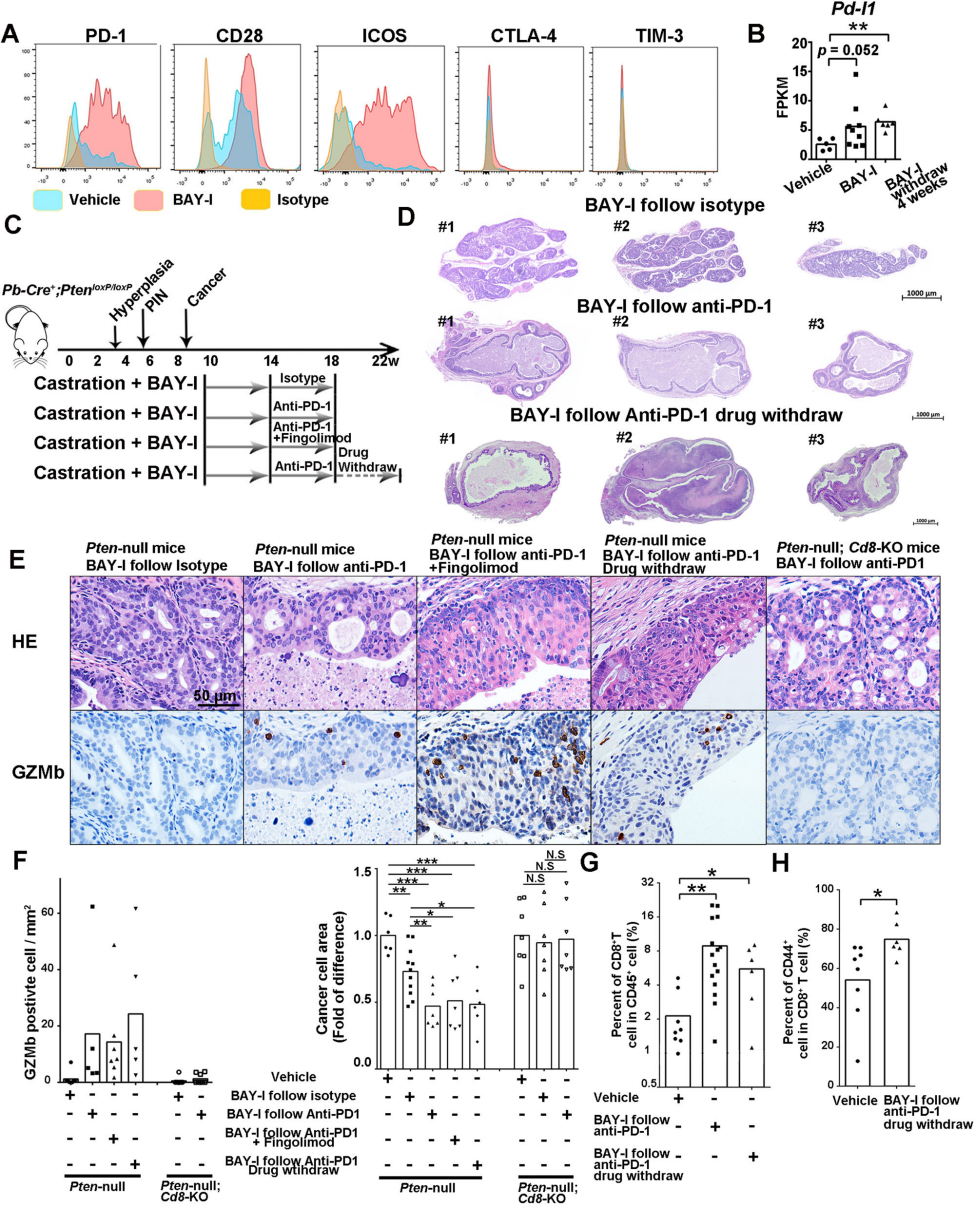
Intermittent BAY1082439 treatment paves the way for subsequent anti-PD-1 therapy and have long-term therapeutic effects. **A** Castrated *Pten*-null mice were treated with 4 weeks of vehicle (*n* = 4) or BAY-I (*n* = 4). Cell surface expression levels of co-inhibitory/stimulatory molecules on tumor-associated CD8^+^ T cell were determined by FACS. **B**. Increased PD-L1 expressions induced by BAY-I treatment. Lin^-^EPCAM^+^ cancer cells from indicated cohorts (*n* = 5 in vehicle cohort, *n* = 9 in BAY-I cohort, *n* = 6 in BAY-I withdraw cohort) were sorted by FACS, and the relative *Pd-l1* expression levels were determined by RNA-seq analysis. Data were presented as mean with dot plots; **p < 0.01 by two-sided T-test. A schematic illustration of treatment strategy. Castrated *Pten*-null mice were treated with vehicle (*n* = 8), or BAY-I for 4 weeks followed by isotype antibody (*n* = 11) or anti-PD-1 antibody (*n* = 15), or anti-PD-1 antibody plus S1PR modulator Fingolimod (*n* = 5) treatment for 4 weeks, or anti-PD-1 antibody then withdrawal for 4 weeks (*n* = 6) (**C**). Low power HE stained images of the anterior prostate lobes from *Pten*-null mice treated with BAY-I follow isotype or anti-PD-1 antibody, or anti-PD-1 antibody then withdrawal for 4 weeks (**D**). The anti-tumor immunity induced by sequential BAY-I and anti-PD-1 combination treatment is not influenced by S1PR modulator Fingolimod and is CD8^+^ T cell dependent. The *Pten*-null and *Pten*-null;*Cd8*-KO mice were treated as indicated in **C**. Tumor tissues were fixed, weighted, and stained with H&E and anti-GZMb antibody. The same staining were performed with 6 mice in all cohort and similar results were observed. (**E**), GZMb^+^ cells density in cancer cell acini (*n* = 8, 5, 7, 5, 7 and 8 in each cohort) and cancer cell areas in the anterior lobes (*n* = 6, 11, 7, 7, 6, 7, 7 and 7 in each cohort) (**F**) were calculated. The percentages of tumor associated CD8^+^ cell in CD45^+^ cell (*n* = 8, 15 and 6 in each cohort) (**G**) and CD44^+^ in CD8^+^ cells (*n* = 8 and 6 in each cohort) (**H**) was determined by FACS. Data were presented as mean with dot plots; N.S *p* > 0.05, *p < 0.05, **p < 0.01, ***p < 0.001 by two-sided T-test. Source data and exact *p* values are provided in the Source Data file.

Cell surface co-expressions of PD-1/CD28 in tumor infiltrating CD8^+^ T cells are positive signs for successful ICT^2,47^. Thus, we investigated the cell surface expression patterns of PD-1 and other co-stimulatory and inhibitory markers in tumor-associated CD8^+^ T cells in the BAY-I treated tumors. FACS analysis revealed that PD-1 and co-stimulatory receptor CD28 and ICOS were upregulated in intratumoral CD8^+^ T cells (Fig. 8A). On the other hand, late exhaustion marker TIM-3 and CTLA-4 were not expressed on the cell surface even through their gene expressions were upregulated (Figs. 7D and 8A), suggesting that the tumor-associated CD8^+^ T cells were not completely exhausted^48^. This notion was further backed by the continuously increased expressions of IL2, TNFα/β and IFNγ in tumor-associated CD8^+^ T cell in BAY-I and BAY-I withdrawal groups (Fig. 7D).

As BAY-I withdrawal tumors had significantly increased IFNγ expression, and IFNγ are known to induce PD-L1 expression^49^, we hypothesized that increased IFNγ expression in BAY-I treated tumor may lead to increased PD-L1 expression in *Pten*-null cancer cells. Indeed, IFNγ treatment significantly increased PD-L1 cell surface expression in the *Pten*-null CAP2 and CAP8 prostate cancer cells (Supplementary Fig. 8A), and RNA-seq analysis on the EpCAM^+^ cancer cells isolated from BAY-I treated and withdrawal group demonstrated that the expression of PD-L1 was significantly increased in these two cohorts (Fig. 8B). Taken together, these results indicated that BAY-I treatment can effectively reprogram CD8^+^ T cells and cancer cells towards favorable response to ICT.

Upregulated PD-L1 expression in prostate cancer cells upon BAY-I treatment may counteract the cytotoxic activity of CD8^+^ T cells, which provided clear rationale for testing anti-PD-1 combination therapy to achieve maximum tumor killing effect. To test this, we first treated castrated *Pten*-null prostate cancer model with BAY-I for 4 weeks, then dosed with control or PD-1 antibody for 4 weeks (Fig. 8C). The mouse body weights did not change significantly (Supplementary Fig. 9A). Anti-PD-1 treatment led to a dramatic cytotoxic effect on otherwise T cell-inflamed tumors, as evidenced by a nearly “hollow” anterior lobe in combinational treatment cohort, which was in sharp contrast to the outcome of BAY-D plus anti-PD-1 group (Fig. 8D, Supplementary Fig. 8B).

Although this sequential combination of BAY-I and anti-PD-1 therapies did not significantly alter the numbers of tumor-associated CD8^+^ and Treg cells (Supplementary Fig. 9B), as compared to BAY-I monotherapy, it did further increased IFNα and IFNγ signaling pathways in tumor tissue (Supplementary Fig. 9C), GZMb^+^ cell (some also co-stained by anti-CD8 antibody) numbers in the remaining tumor areas (Fig. 8E, F and Supplementary Fig. 9D), consistent with increased anti-tumor immunity.

The remarkable effects of sequential BAY-I and ICT treatment observed in our study were dependent on intratumoral CD8^+^ T cells, as co-administration of Fingolimod with anti-PD-1 antibody did not affect the therapeutic effect of anti-PD-1 antibody (Fig. 8E, F). To demonstrate the cytotoxic effect of anti-PD-1 antibody treatment depends on CD8^+^ T cells, we treated *Pten*-null;*Cd8-*KO mice with the same sequential BAY-I and anti-PD-1 combination. The treated *Pten*-null;*Cd8-*KO prostates had almost no GZMb^+^ cells in the cancer areas (Fig. 8E, F). Sequential BAY-I and anti-PD-1 combination treatment significantly decreased cancer cell areas than isotype treated cohort, while this combination effect was absent, in the *Pten*-null;*Cd8-*KO prostate (Fig. 8F).

### Sequential combination of BAY-I and anti-PD-1 therapy leads to long-term durable anti-tumor immunity even after anti-PD-1 antibody withdrawal

As successful immunotherapy often associated with long-lasting durable therapeutic effects^50^, we investigated the potential long-term effect of sequential combination of BAY-I and anti-PD-1 therapy. We withdrew anti-PD-1 antibody in a cohort of mice that had undergone sequential BAY-I and anti-PD-1 combination therapy as shown in Fig. 8C, and observed that the “hollow” anterior lobes and reduced cancer cell areas remained the same (Fig. 8D–F), indicating that the tumor cells failed to rebound. Even though the tumor tissues had high P-AKT and Ki67 staining, IHC and FACS analyses showed persistent infiltration of CD8^+^ T and GZMb^+^ cells (Fig. 8F, G and Supplementary Fig. 9E). FACS analysis also demonstrated that tumor associated CD8^+^ T cell had CD44^+^ memory T cell phenotype (Fig. 8H). Together, these results indicated that a sequential combination of BAY-I and anti-PD-1 therapy could lead to a long-lasting, durable immune cell-mediated anti-tumor effect even after complete drug withdrawal.

## Discussion

We demonstrate in this study that a carefully designed isoform-specificity and dosing schedule for PI3K inhibitor, and sequential administration of targeted and anti-PD-1-mediated ICT can effectively overcome resistance to ICT in a preclinical prostate cancer setting and achieve a long-last durable immune cell-mediated therapeutic effect even after drug withdrawal.

We find in this study that PI3K inhibition by an anti-PI3Kα/β/δ inhibitor, BAY1082439, not only inhibits PTEN null prostate cancer cell growth, but also promotes anti-tumor immunity in the endogenous *Pten*-null prostate cancer model. The in vivo effects of BAY1082439 on cancer cells and tumor microenvironment are intrinsically linked and are associated with its potent anti-PI3Kα/β/δ activities. BAY1082439′s anti-PI3Kα/β activities not only inhibit PTEN null prostate cancer cells growth and prevent feedback activation between PI3K isoforms^51^, but can revert PI3K-dependent immunosuppressive activity by upregulating IFN signaling pathway activity, CXCL10 and CCL5 excretion and B2M expression. These are cancer cell intrinsic property and are reversible upon BAY1082439 withdrawal^30^ (Fig. 1 and Supplementary Fig. 2). Meanwhile, BAY1082439′s anti-PI3Kδ activity can preferentially inhibit tumor-associated Tregs and B cells, alleviating Treg-mediated immunosuppression and lymphotoxin-mediated CRPC growth^30,52^ (Fig. 3 and Supplementary Fig. 4B and Supplementary Table 3), and consequently activating cytotoxic CD8^+^ T cells. It is the combined effects of BAY1082439 on *Pten*-null cancer cells and immune cells in the tumor microenvironment leads to tumor burden reduction as well as tumor-associated CD8^+^ T cell activation, clonal expansion and infiltration into the tumor acini. IFNγ secreted by T cells further activate PDL-1 expression in the cancer cells, which pave the way for anti-PD-1 therapy (Fig. 8B and Supplementary Fig. 8A).

Our study showed that not only the isoform profile but also the dosing schedule of BAY1082439 is critically important for promoting anti-cancer immunity. Intermittent but not daily BAY1082439 treatment can turn “cold” *Pten*-null prostate cancers to T cell-inflamed (Figs. 2 and 3). Although both daily and intermittent dosing of BAY1082439 can effectively inhibit PTEN-null prostate tumor cell growth and decrease the number of tumor-associated B cells and Tregs, only intermittent dosing schedule can activate the intratumoral cytotoxic CD8^+^ T cells, allow them to undergo clonal expansion, and infiltrate into the cancer acini probably via increased CCL5 or CXCL10 secretion (Figs. 2, 3, 4, 6 and 7). The differential effects of daily vs. intermittent dosing of BAY1082439 on Treg and CD8^+^ T cells are most likely due to the different sensitivities of these immune cells to the anti-PI3Kδ inhibitor, similar to previous reports^39,41^. As Tregs are most sensitive to BAY1082439, intermittent dosing is sufficient to reduce tumor-associated Treg number and alleviate its immunosuppressive activity, which provides a window for CD8^+^ T cell activation and clonal expansion, as indicated by RNA-seq analysis of isolated CD8^+^ T cells (Figs. 4 and 7). Intermittent treatment could also minimize aberrant immune activation in non-cancerous organs, avoiding adverse side-effects (Supplementary Fig. 3). Intermittent dosing of PI3K inhibitors have been reported by other works and shown to reach successful therapeutic effect while improve drug tolerance^35,53,54^. Noticeably, other targeted therapeutic inhibitors have similar inhibitory effects on CD8^+^ T cells, such as androgen receptor antagonists^55^ and BRAF/MEK inhibitors^56^. Optimization the dosing schedule of these inhibitors may also improve their therapeutic effects as monotherapies or in combination with ICT.

Intermittent BAY1082439 treatment triggers tumor-specific CD8^+^ T cell activation and expansion, most likely through intra-tumoral TLS, as pre-treating the *Pten*-null CRPC model with S1PR modulator Fingolimod to block lymphocyte egress from hematopoietic organs and lymph nodes^42^ cannot prevent intra-tumoral CD8^+^ cell activation, clonal expansion and anti-tumor immunity (Figs. 4, 6 and 8). A recent report demonstrates a cloning replacement of tumor-specific T cells following ICT in human basal or squamous cell carcinoma, and suggest that pre-existing tumor-specific T cells may have limited role in ICT^57^. The different conclusions on the origins of tumor-specific T cells in our study and those by Yost et al.^57^ may due to the unique characteristics of the cancer type or the specific time windows-associated with each study.

We demonstrate that the CD8^+^ T cells play an essential role in BAY1082439-induced anti-tumor immunity. Importantly, genetically deleting or antibody-mediated depleting CD8^+^ T cells can almost completely diminish the therapeutic effect of BAY1082439-induced anti-tumor immunity (Figs. 5 and 8). However, other immune cells may also contribute to the overall therapeutic outcome. We and others have demonstrated the contribution of B cells in CRPC development^30,52^ and we have shown that BAY1082439 could significantly inhibit B cells in the *Pten*-null CRPC model^30^. Similarly, MDSC is increased and DC maturation is decreased in the *Pten*-null model^18,20^ and the effects of BAY1082439 intermittent treatment on MDSC and DC maturation need further investigation. Together, the superior effects of intermittent BAY1082439 treatment support the idea that drugs that can co-target both cancer cell-intrinsic and microenvironment pathways may have considerably more clinical benefit than single-target drugs.

Recent clinical studies revealed that, depending on the CD8^+^ T cell infiltration levels, solid tumor can be divided into T cell-inflamed “hot tumor” or non-T cell inflamed “cold tumor”^5^. Treatments that can improve T cell infiltration may augment ICT efficacy^2,5,58^. Here we show that intermittent BAY1082439 treatment can turn PTEN-null prostate tumor from “cold” to T cell-inflamed (Fig. 2). Intriguingly, once the tumor has become T cell-inflamed, it stays in T cell-inflamed status even after drug withdrawal (Figs. 6–8). The CD8^+^ T cells remain in the tumor acini, carry memory T cell signature and are not completely exhausted, while the tumor cells have up-regulated antigen-presentation and high PD-L1 expression (Figs. 6–8). This T cell-inflamed state paves the way for successful anti-PD1 treatment. Importantly, the T cell-inflamed state and memory T cell signature remain even after 4 weeks of anti-PD1 antibody withdrawal, demonstrating long-last and durable anti-tumor immunity (Fig. 8). Although the detailed mechanisms associated with this prolonged response require further investigation, our study provides a successful pre-clinical case for sequential, instead of simultaneous, anti-PI3K and ICT combination treatment to avoid potential combined toxicity when both drugs are used together.

Results from our study are consistent with multiple previously published works, which demonstrate that: (1) PTEN-null CRPC cancer cells are sensitive to PI3K-AKT axis inhibition^17,59,60^; (2) Efficient targeting PI3K in cancer cell need balanced PI3Kα/β inhibitor to avoid feedback activation between PI3K isoforms^51^; and (3) Targeting PI3K isoform δ or α/δ can break Treg-mediated immune suppression and activate CD8^+^ T cells^39,41,61^. On the other hand, prolonged systematic PI3K inhibition may also hinder the anti-tumor activity of CD8^+^ T cell^29,62^, implying the need for intermittent treatment and sequential PI3K and anti-PD-1 therapeutic strategy. Besides our research on genetically engineered mouse models, a recent research also showed that in transplantable models, a PI3Kα/δ inhibitor had CD8^+^T cell promoting effect on a 5on/2off dosing schedule^61^. Future works are need to test the effects of BAY-I and sequential BAY-I and anti-PD-1 treatment strategies on other models with different cancer-initiating and immune evasion mechanisms, and in humanized mouse cancer models before moving to clinical settings.

In summary, our results demonstrate that a carefully designed anti-PI3K treatment, both in its specificity and dosing schedule, to inhibit cancer cell growth while promote anti-tumor immunity, is critically important for successful combination of anti-PI3K targeted therapy and ICT. Since PTEN loss or PI3K pathway activation is one of the most frequently altered pathways in human cancers, our work may shed light on how to successfully combine anti-PI3K targeted therapy with ICT for broad and long-lasting therapeutic effects on other cancer types with PTEN loss or PI3K activation.

## Methods

### Animals

*Pb-Cre*^*+*^*;Pten*^*loxP/loxP*^ *(Pten*-null) mouse model and castration procedure were described previously^15^. All animal housing, breeding and experimental procedures was approved by the Ethics Committee of the Peking University with ID under LSC-WuH-1. *Cd8*^*atm1Mak*^ mice^44^ (*Cd8*-KO mice) was purchased from the Jackson lab (002665) then crossed with the *Pten*-null mice to generate *Pb-Cre*^*+*^*;Pten*^*LoxP/LoxP*^*;Cd8*-KO (*Pten*-null;*Cd8*-KO) mice. *Pb-Cre*^*+*^*;Pten*^*LoxP/LoxP*^*;K-ras*^*G12D/W*^ mice model were described previously^38^. For all animal experiments, the animals were monitored carefully, and no body-weight loss exceeds 20% in all treatment cohorts.

For surgical castration, mouse was firstly anaesthetized by avertin. Briefly, 2,2,2-Tribromoethanol (Sigma-Aldrich, T48402) was dissolved in tert-amyl alcohol (Sinopharm Chemical ReagentCo, 8007828) to 1.6 g/mL as stock solution, then further dilute to 20 mg/mL in saline as the working solution. Animals were injected intraperitoneally at 250 mg/kg per mouse. Then, a midline ventral skin incision was made into the scrotum and testicles were removed from both sides by sealing off the blood vessels using suture line (Jinhuan Medical, CR537). The skin incision was then sutured using suture line. The animals were closely monitored during the procedure for signs of pain or bleeding and placed in a clean cage for post-surgery recovery.

### Cell culture

The CaP2 and CaP8 cell lines were established as described previously^16^ and cultured in DMEM media (Gibco 11995065) containing: FBS (10%, Applied StemCell B7227), Penicillin-Streptomycin (1%, Gibco 15070063), L-Glutamine (1%, Gibco A2916801), Insulin (5ug/ml, Gibco 12585-014), EGF (6 ng/ml, Gibco PHG0313), Bovine Pituitary Extract (25ug/ml, Gibco 13028-014). The PC3 and LNCaP cell lines were purchased from ATCC and the PC3 PTEN inducible cell line are generated as describe^17^ and cultured using Tet-free serum (Omega Scientific FB-15). All cell was cultured at 37 °C in 5% CO^2^ condition. PTEN re-expression was induced by adding Doxcycline (1 ug/ml, sigma D9891) in culture media for 4 days. IFNγ (PEPROTECH, 315-05) was add at 10IU concentration for 72 h, PDL1 expression in CAP2/CAP8 cell was determined by FACS.

### Elisa detection of secreted cytokines

CaP2 and CaP8 cells (1 × 10^5^) were seed in 6-well plate, cultured in media containing solvent or 5 μmol BAY1082439 (dissolved in DMSO with 10mmolTFA as 5 mM stock solution). Total cell number was counted using cell counter 48 h later and supernatant was harvested. Murine CCL5 and CXCL10 concentrations were measured by using Elisa kit R&D MMR00 and Cusabio CSB-E08183h, respectively, according to manufacture recommendations.

### Inhibitors and reagents

Ipatasertib (AKT inhibitor) and BAY1082439 was provided by Bayer AG. S1P receptor modulator Fingolimod (FTY720), and BrdU was purchased from MCE. TGX221 (PI3Kβ inhibitor) and CAL101(PI3Kδ inhibitor) was purchased from Selleck.

### Western blotting

Cultured cells were washed 3 times in PBS (Thermo fisher,14040117) then lysed with RIPA buffer (Beyotime, P0013B) containing Protease & Phosphatase Inhibitor (Thermo fisher, A32959), the protein concentrations were then determined by using BCA Kit (Thermo Fisher Scientific, 23227). Proteins were separated by SDS-PAGE then transferred to PVDF membrane (Merck Millipore, ESEQ00010). The membrane was blockaded by 5% BSA (Amresco, E588-25g) in TBST buffer then incubated with primary antibodies in 5%BSA-TBST buffer overnight at 4 °C, washed in TBST buffer for 3 times then incubated with secondary antibody in TBST for 1 h at room temperature. The signal was detected by using ECL western blotting substrate kit (Thermo fisher, 32209 and 34095). The following primary antibodies were used: Mouse anti-Mouse/human anti-β-actin antibody (Zsbio, TA-09; 1:1000), Rabbit anti-Mouse/human pan-AKT antibody (Cell Signaling Technology, 4691; 1:1000), Rabbit anti-Mouse/human phosphate AKT(Ser473) antibody (Cell Signaling Technology, 4060; 1:1000), Rabbit anti-Mouse B2M antibody (Cell Signaling Technology, 59035; 1:1000), and Rabbit anti-Mouse/Human PTEN antibody (Cell Signaling Technology 9188; 1: 1000). The following secondary antibodies were used: HRP-conjugated anti-mouse antibody (Jackson ImmunoResearch Laboratories, 115-035-003; 1:5000). HRP-conjugated anti-rabbit antibody (Jackson ImmunoResearch Laboratories, 113-035-003; 1:5000). Image Lab 5.0 software are used for data collection and analysis. All FACS antibodies used are listed in Supplementary Table 2.

### Tissue Dissociation, Single-cell Suspension and FACS analysis

Prostates were dissected and photographed, weighted and minced in sterile tissue culture dishes, and subjected to collagenase A (1.5 mg/ml; Roche) and DNase I (0.1 mg/ml; Roche) digestion for 1 h at 37 °C with constant agitation. Undigested tissue was removed by passing a 70-μm filter and the total cell number was counted using cell counting chamber. The single-cell suspensions were firstly stained with Fixable Viability Stain 450 (BD) then with fluorescent-labeled antibody against CD45 (Biolegend, 103105 or 103147 or 103108; 1:100), CD11b (Biolegend, 101206; 1:200), Gr-1 (Biolegend, 108411; 1:100), CD3 (Biolegend, 100219; 1:100), CD8 (Biolegend, 100713; 1:100), CD4 (Biolegend, 100429; 1:200), CD25 (Thermo fisher, 17-0251-82 1:100), PD-1 (Biolegend, 135209; 1:100), Tim-3 (Biolegend, 119721; 1:100), CTLA-4 (Biolegend, 106305; 1:100), CD28 (Biolegend, 102105; 1:100), CD44 (Biolegend, 103047; 1:100), CD62L (Biolegend, 104405; 1:200) or ICOS (Biolegend, 107705; 1:100). FOXP3 (Thermo fisher, 12-4771-82; 1:100) was stained by using eBioscience™ FOXP3 Staining Buffer Set (Thermo fisher, 00-5523-00). Sorting of each T cell population was performed using BD FACSAria™ III. Different cell population was defined as: CD8^+^ T cells: CD45^+^CD3^+^CD8^+^; CD4^+^ T cells: CD45^+^CD3^+^CD4^+^; Treg: CD45^+^CD3^+^CD4^+^CD25^+^FOXP3^+^. Data was analyzed by using flowjo software. All FACS antibodies used are listed in Supplementary Table 2 and all gating strategies are presented in Supplementary Fig. 10. BD FACS Diva 8.0 software was used to collect data and data analysis are performed in Flowjo 5.0 software.

### In vitro T cell culture

Single-cell suspensions from mice spleen were first stained with Fixable Viability Stain 450 (BD 562274) and CFSE (Thermo C34554) to label live and proliferated cells, followed by fluorescent-labeled antibody against CD45 (Biolegend, 103105; 1:100), CD8 (Biolegend, 100713; 1:100), CD4 (Biolegend, 100429; 1:200), CD25 (Thermo fisher, 17-0251-82 1:100) or CD127 (Biolegend 135025; 1:100). Viable CD8^+^ T cells (CD45^+^CD8^+^), helper T cells (CD45^+^CD4^+^CD25^-^) and Tregs (CD45^+^CD4^+^CD25^+^CD127^low/-^) from single-cell suspension were sorted by FACS. Cells were seeded at a density of 2.5 × 10^4^/well in 96-well plates pre-coated with 10ug/ml CD3 (Biolegend,100339;1:100) and 1ug/ml CD28 (Biolegend, 102115; 1:1000) antibodies and cultured in 1640 media with 10% FBS, NEAA (Thermo 11140050), 1 mM Sodium Pyruvate (Thermo 11360070), 50 umol β-ME and 100 IU/ml IL2^63^, as well as different concentrations of inhibitors or solvent control. Four days later, cells were harvested, live cell numbers were counted by cell counter, and T cell proliferation rate (proliferated cell was determined by CFSE staining) was determined by FACS. Cell growth rate was calculated by the percentage of live, proliferated cell number at each drug concentration vs. solvent control.

### In vivo drug treatment

BAY1082439 was dissolved in 0.1 N HCL at 18 mg/ml and orally administered. For Single bullet treatment, BAY1082439 was administered at a dose of 180 mg/kg/d and mice were analyzed 24 h later. For daily treatment, BAY1082439 was administered at a dose of 75 mg/kg/d for 4 weeks. For intermittent treatment, BAY1082439 was administered at a dose of 180 mg/kg/d in a 2 days on/5 days off manner for 4 weeks. S1PR modulator (Fingolimod, FTY720) was dissolved in 0.9% NaCl saline solution and orally administered at 1 mg/kg/d. Anti-PD-1 (BioXcell, BE0146) or isotype control (BioXcell, BE0089) antibody (200ug per mice) was dosed 3 times per week by i.p. Anti-CD8 (BioXcell, BP0061) antibody (200ug per mice) was dosed 3 times per week by i.p. BrdU was dissolved in PBS at 10 mg/ml and dosed 100 mg/kg by i.p 24 h before analysis. BrdU positive cells were analyzed by Flow Cytometry using eBioscience™ BrdU Staining Buffer Set (00-5525-00) and anti-BrdU antibody (Biolegend 339812 1:100).

### Histology and IHC Analysis

H&E, immunohistochemistry (IHC), and immunofluorescence (IF) staining were performed as described previously^15^. In brief, the slide was firstly dewaxed in 100% Xylenes (Beijing Tong Guang Fine Chemistry 102218) for 15 min and repeat 2 times, then hydrated in 100% Ethanol (Beijing Tong Guang Fine Chemistry 104021) for 10 min and repeats 2 times then sequentially in 95%,85%,70%, 50% Ethanol and water for 2 min. the antigen retrieval was performed by incubating the slides in 0.01 M citric acid buffer (pH 6.0) at 95 °C for 20 min then let the slides cool-down naturally to room temperature in citric buffer. The slide was washed in PBS (pH 7.4) for 10 mins three times then incubated in 3% H_2_0_2_-PBS solution (ZSGB-BIO ZLI-9311) to remove endogenous peroxidase activity, then washed in PBS again for 3 times, blocked in in 10% goat serum (ZSGB-BIO ZLI-9022) for 30 min, then stained with antibodies at dilutions provided in manufacturer’s protocol. For IHC, the subsequent procedure was performed by using goat two-step signal detection kit (ZSGB-BIO PV-9001) under manufacturer’s protocol. For IF, the slide was washed in PBS again, incubated in PBS containing immunofluorescence fluorescent secondary antibody, then finalized by using Mounting Medium with DAPI (abcam 104139). The antibodies used and dilutions were listed in Supplementary Table 2. OLYMPUS cellSens standard software (version 1.11) was used for IHC and IF image capture. All antibodies used are listed in Supplementary Table 2.

To calculate cancer cell area, HE stained slides was scanned, anterior lobe was identified and (1) superimposed each image with grids; (2) marked those grids with at least 30% area within cancerous glandular structure, and eliminated those samples with < 5 grids; (3) calculated cancer cell areas in each grid using Zen 2.3 software, and then combined all grids for total cancer cell areas; (4) divided total cancer cell areas by the grid number to generate cancer cell area/grid; and (5) calculated fold of changes between treatment cohort and vehicle cohort.

### Real time PCR

RNAs were extracted from tumor tissues or cancer cell lines then reverse transcribed using Kit from RNAeasy mini kit (Qiagen 74106) and Vazyme (R223-111 01). Quantitative PCR was achieved using Invitrogen SYBR mix. Primers were listed in Supplementary Table 2. Real-Time PCR data collection was performed by using Bio-Rad CFX manager 3.1 software.

### RNA-seq analysis

For RNA-seq analyses, bulk prostate tissues, sorted LIN^-^EpCAM^+^ prostate cancer cells and cancer- or spleen-associated CD45^+^CD3^+^CD8^+^ T cells were used. RNA was extracted by using RNAeasy mini kit (Qiagen 74106) or micro kit (Qiagen 74004), and cell line or tumor tissue cDNA library was constructed by using NEBNext® Poly(A) mRNA Magnetic Isolation Module (E7490) and NEBNext® Ultra™ RNA Library Prep Kit for Illumina® (E7530). The cDNA library of CD8^+^ T cell and cancer cell was constructed by using SMART-SEQ 2 protocol. Sequencing was performed by Illumina - HiSeq PE150.

All RNA-seq data were aligned to the mm10 genome using Tophat (version v2.1.1). Differentially expressed genes were identified by Cuffdiff (version v2.2.1)^64^. FPKM was used for following analysis and comparison. GSEA analysis was performed as software suggested^65^. T cell clonotype diversity analysis was performed by TRUST^43^. The pathway activity score was calculated with GSVA^66^. The expression levels of TLS score genes^46^ were scaled to the range of 0 to 1, and TLS score was defined as the mean scaled value of related genes for each individual sample,

### Analysis of human prostate cancer samples

The relationships between IFNα/γ score and CCL5, CXCL10, B2M and CD8A gene expression in human prostate, lung and melanoma cancer tissues were analyzed by using data from cBioPortal^67^ (Prostate Adenocarcinoma: Firehose Legacy datasets (499 patients)); Lung Adenocarcinoma: TCGA PanCancer Atlas datasets (566 patients); Skin Cutaneous melanoma: TCGA PanCancer Atlas datasets (448 patients).

### Statistical analysis

GraphPad Prism software was used to calculate means and SEM/SDs and Pearson correlation coefficient. Gene Set Enrichment Analysis was performed by GSEA. The Student *t* test in excel 2019 software was used to determine statistical significance, and *p* < 0.05 (two-sided) was considered statistically significant.

### Reporting Summary

Further information on research design is available in the Nature Research Reporting Summary linked to this article.

## Supplementary information

Supplementary information

Reporting Summary

Description of Additional Supplementary Files

## Data Availability

Source data are provided with this paper. All patient RNAseq data analyzed are available at TCGA database and from cBioPortal (https://www.cbioportal.org/). All RNAseq raw data used in Figs. 1, 2, 4, 6, 7, Supplementary Figs. 2, 6, 9 and Supplementary Data 1 are available at Gene Expression Omnibus under accession number GSE159660. The remaining data are available within the Article, Supplementary Information or Source Data file. Source data are provided with this paper.

## Source data

Source Data
