# Supplementary material for: Overcoming resistance to immune checkpoint therapy in PTEN-null prostate cancer by intermittent anti-PI3Kα/β/δ treatment
Source: Nat Commun. 2022 Jan 10;13:182. doi: 10.1038/s41467-021-27833-0 (PMC8748754; doi:10.1038/s41467-021-27833-0)
Supplement: Supplementary file 1 — Supplementary information [file 41467_2021_27833_MOESM1_ESM.pdf]

Overcoming resistance to immune checkpoint therapy in PTEN-null prostate cancer by sequential intermittent anti-PI3Kα/β/δ and anti-PD-1 treatment

Zhi Qi[1,2,3], Zihan Xu[2], Liuzhen Zhang[1,2,3], Yongkang Zou[1,2,3,4], Jinping Li[1,2,3], Wenyu Yan[1,2,3], Cheng Li[2], Ningshu Liu[5#] and Hong Wu[1,2,3,4*]

[1]The MOE Key Laboratory of Cell Proliferation and Differentiation, [2]School of Life Sciences, [3]Peking-Tsinghua Center for Life Sciences, Peking University, Beijing 100871, China; [4] Institute for Cancer Research, Shenzhen Bay Laboratory, Shenzhen 518107, China; [5]Bayer AG, Drug Discovery TRG Oncology, Muellerstrasse 178, 13353 Berlin, Germany

[#]Current address: Hehlius Biotech, Inc., 1801 Hongmei Rd, Shanghai 200233, China

Supplementary Figures and Figure legends

Supplementary Figure 1

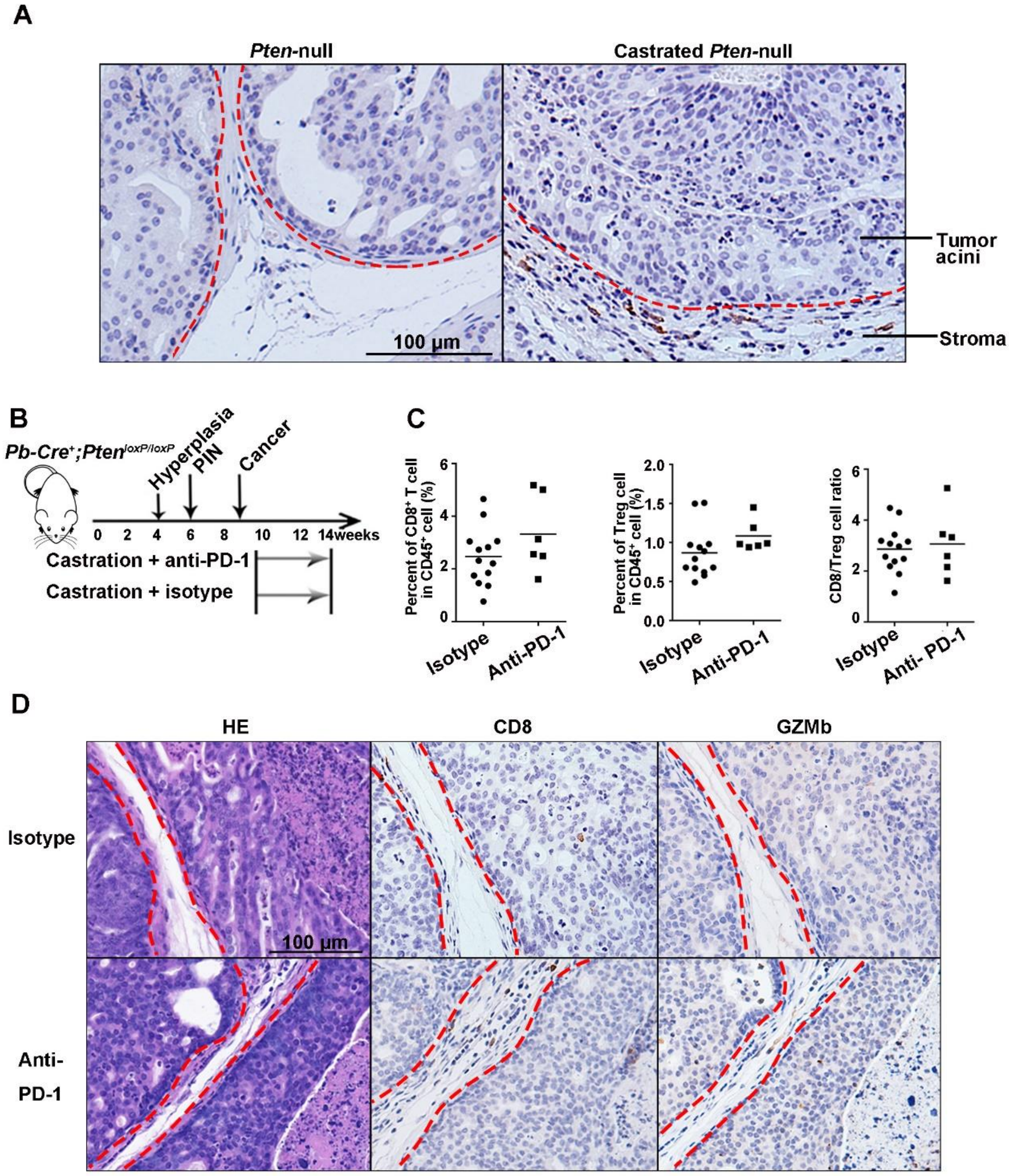

Supplementary Figure 1. *Pten*-null prostate cancers have low T cell infiltration and are resistant to anti-PD-1 monotherapy

A. Immunohistochemistry (IHC) analysis shows low $CD8^+$ T cell infiltration in primary (left) and CRPC (right) *Pten*-null prostate cancer model. The same staining was performed with 6 mice in each cohort and similar results were observed.

B-D. A schematic illustration of treatment strategy (B), the percentages of tumor-associated $CD8^+$ in $CD45^+$, Treg in $CD45^+$ and $CD8^+$/Treg ratios after isotype (n=13) or anti-PD-1 (n=6) antibody treatment for 4 weeks (C), and H&E and IHC analyses for infiltrating $CD8^+$ and $GZMb^+$ cells after each treatment (D), The same staining was performed with 6 mice in each cohort and similar results were observed..

Red dash line marks the boundary between cancer acini and stroma areas. Data in C were presented as dot plots with mean as central lines. Source data and exact *p* values are provided in the Source Data file.

Supplementary Figure 2

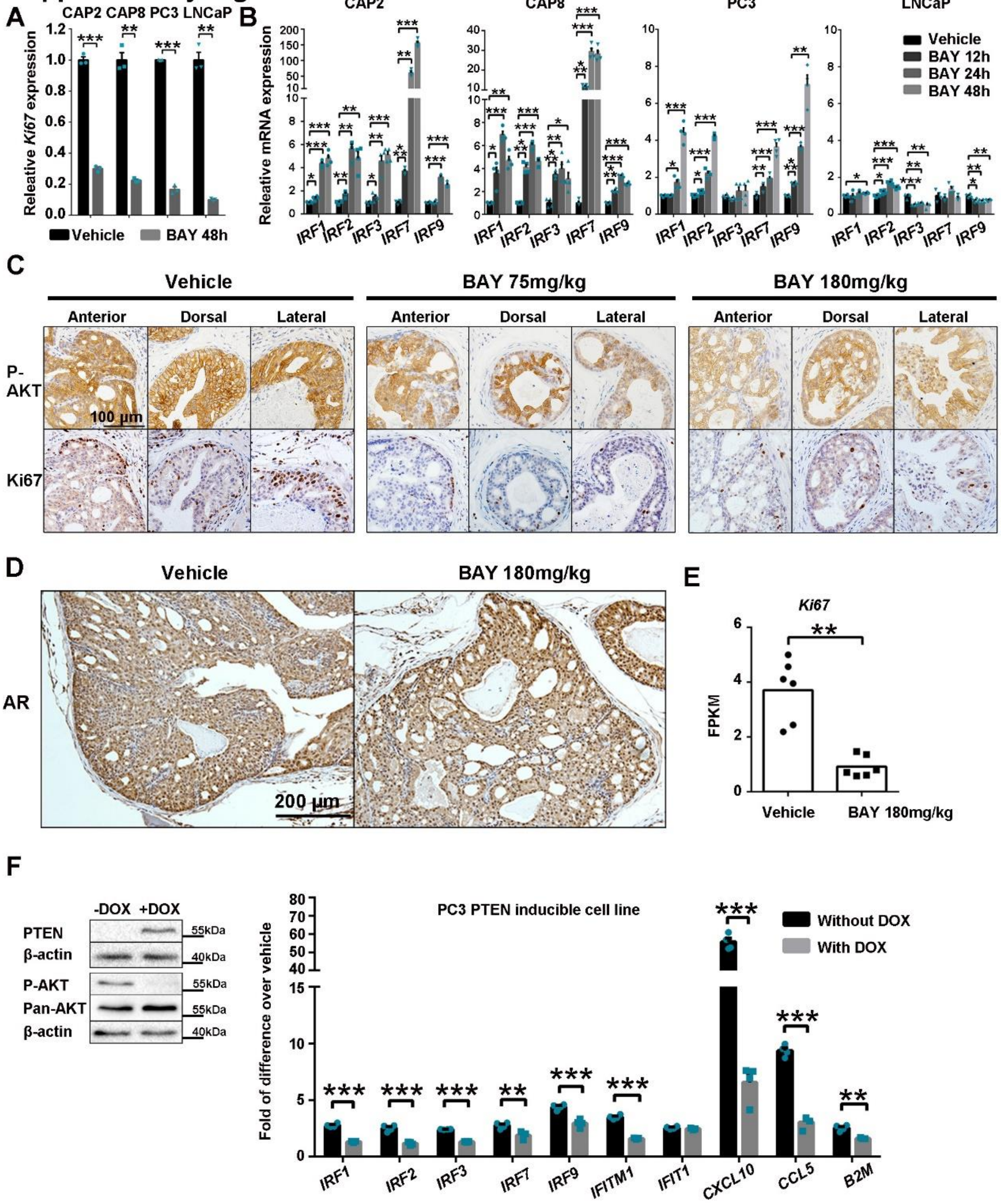

Supplementary Figure 2 - continued

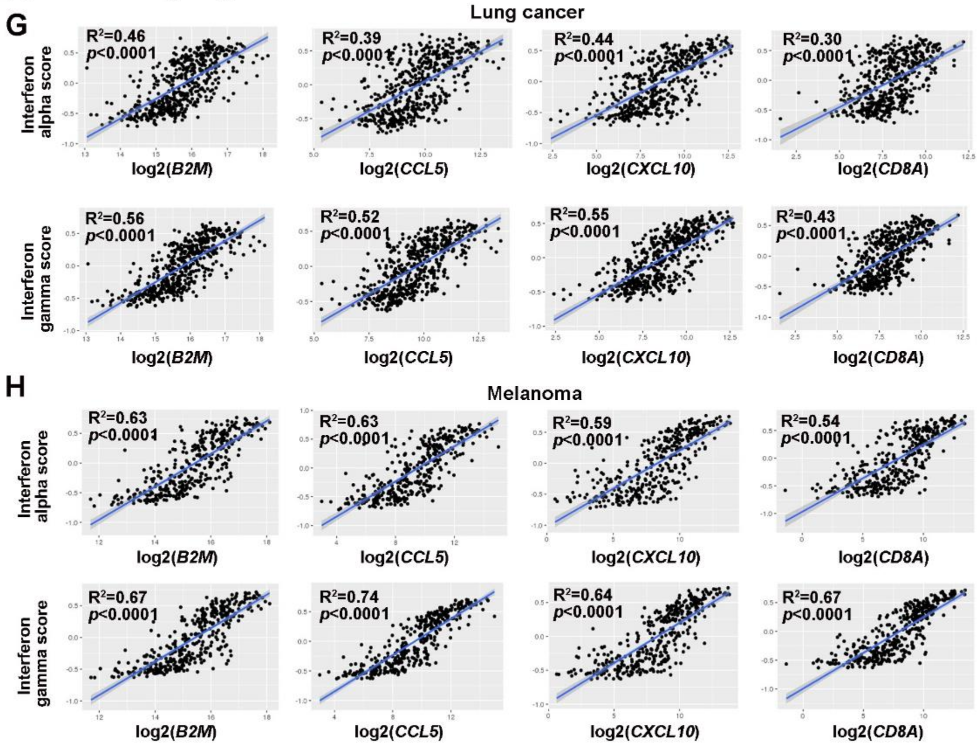

Supplementary Figure 2. BAY1082439 treatment inhibit cancer cell proliferation and alleviates cancer cell-intrinsic immunosuppressive activity

A. PTEN null CAP2, CAP8, PC3 and LNCaP prostate cancer cell lines were treated with vehicle (n=3) or 5μM BAY1082439 (n=3) for 48h. RNA-seq analyses were performed, the relative expression of *Ki67* gene was analyzed. ** $p<0.01$, *** $p < 0.001$ by two-sided T-test. Data were represented as mean+SEM.

B. Time-dependent responses of BAY1082439 (5μM) treatment in four PTEN null prostate cancer cell lines. Relative mRNA expressions were measured by RT-PCR analysis and data were represented as mean±SEM of fold of changes between BAY and vehicle. n=4 for each cell lines and treatment condition. * $p<0.05$; ** $p<0.01$, *** $p < 0.001$ by two-sided T-test.

C-E. The effects of a bullet dose BAY1082439 treatment on cancer cell P-AKT level,

AR level and cell growth**.** *Pten*-null mice were treated with vehicle (n=6), BAY1082439 75mg/kg/day (n=3) or BAY1082439 180mg/kg/day (n=6) for 2 days, then tumor tissues were harvested 4 or 24h later, respectively. Immunohistochemistry was performed to assess Ki67, P- AKT (C) and AR levels (D), The same staining were performed with 6 mice in each cohort and similar results were observed. RNAs were extracted for RNA-seq analysis and *Ki67* expression levels were compared (E). **, $p<0.01$ by two-sided T-test.

F. PTEN-dependent BAY1082439 responses in PC3 cells. PTEN was re-expressed in PTEN inducible PC3 cells by doxycycline induction (left). Relative mRNA expressions were measured by RT-PCR analysis without and with PTEN re-expression, and data were represented as mean±SEM of fold of changes between BAY and vehicle. n=4 for each cell lines and treatment condition. ** $p < 0.01$, *** $p < 0.001$ by two-sided T-test.

G-H. The positive correlations between IFNα/γ activity scores and *CCL5/CXCL10/B2M/CD8A* gene expressions in the human lung (566 patients) (C) and melanoma (448 patients) (D) cancer tissues. Linear regression was used, error bands represent 95% confidence intervals. Statistical test done by two-sided T test. Source data and exact *p* values are provided in the Source Data file.

Supplementary Figure 3

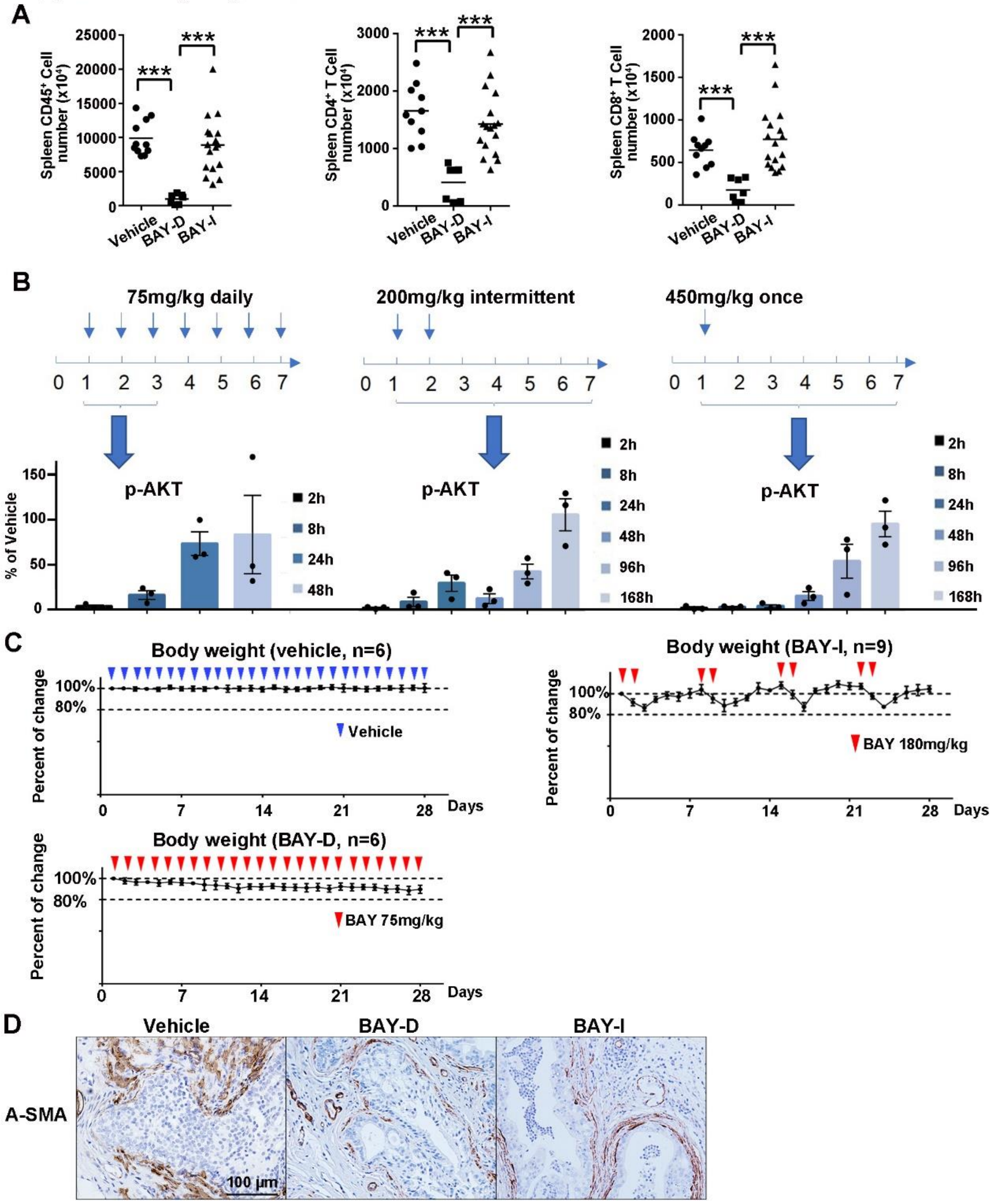

Supplementary Figure 3. The effects of BAY1082439 treatment on cancer cell and immune landscape (1)

A**.** The effects of Vehicle (n=11), BAY-D (n=7) and BAY-I (n=17) treatment on spleen-associated $CD45^+$, $CD4^+$ and $CD8^+$ cells. Data were based on FACS analysis and presented as dot plots with mean as central lines. ** p<0.01, *** p < 0.001 by two-sided

T-test.

B. The effects of different BAY1082439 dosing schedule on P-AKT levels in GXA3027 PDX model. BAY1082439 was dosed at 75 mg/Kg daily, 200mg/Kg intermittent or 450mg/kg once, for a week, each indicated time point have 3 mice In both vehicle or drug treated cohort. Tumor tissues were taken at 2, 8, 24, 48, 96 and 168 hr post the 1st dosage and P-AKT levels were measured. Data were represented as mean $\pm$ SEM.

C-D. The effects of BAY-D or BAY-I treatment on mouse body weight and pathology. Castrated *Pten*-null mice were treated with vehicle, BAY-D or BAY-I for 4 weeks and the mouse body weights were recorded as indicated (C); Prostates were fixed and Immunohistochemistry was performed for anti-SMA (D). The same staining were performed at 7 mice in each cohort and similar results were observed. Source data and exact *p* values are provided in the Source Data file.

Supplementary Figure 4

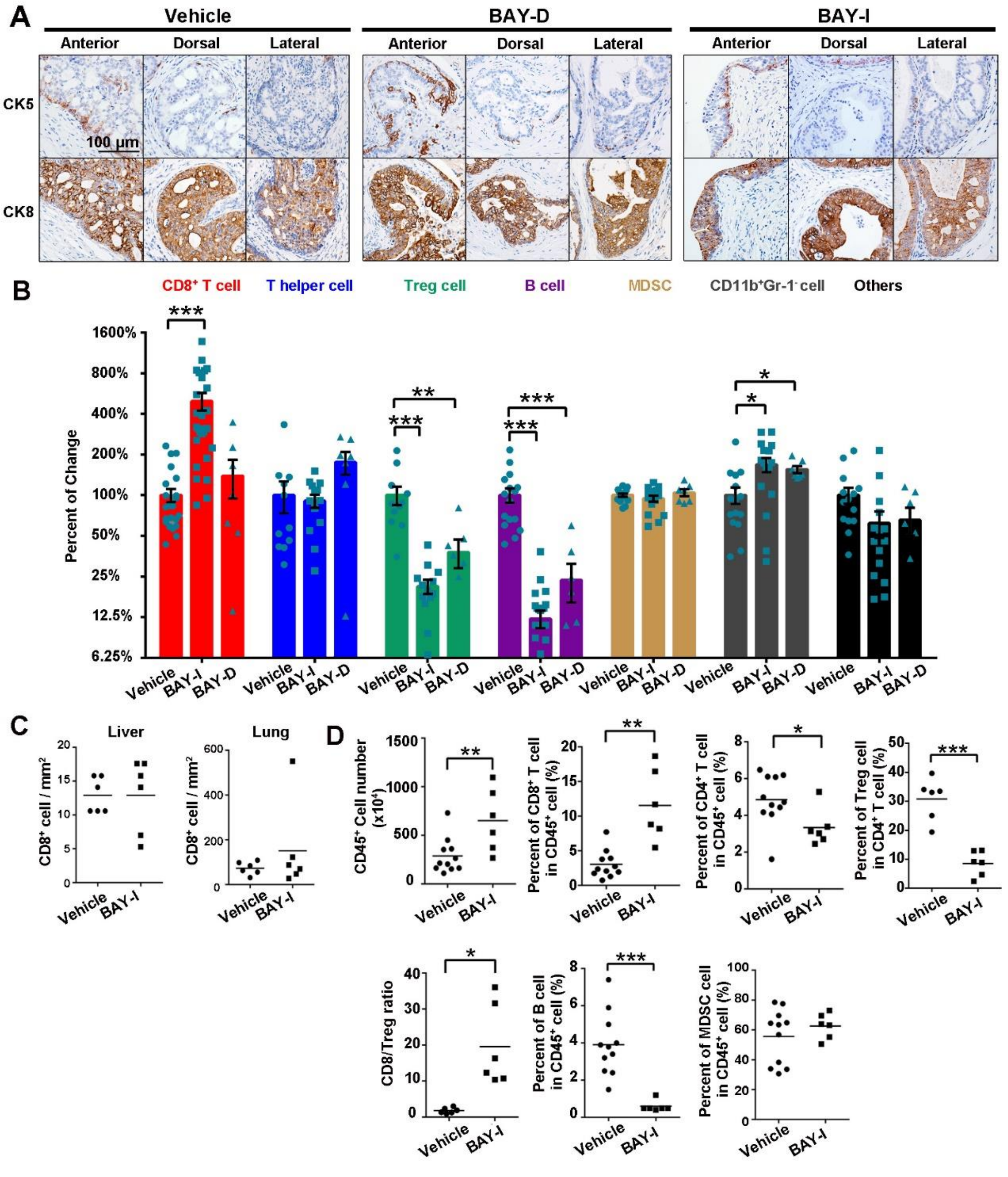

Supplementary Figure 4. The effects of BAY1082439 treatment on cancer cell and immune landscape (2)

A. The effects of BAY-D or BAY-I treatment on mouse prostate cancer cell. Castrated *Pten*-null mice were treated with vehicle, BAY-D or BAY-I for 4 weeks. Prostates were fixed and Immunohistochemistry was performed for CK5 or CK8 antibodies. The same

staining were performed with 6 mice in vehicle and BAY-I cohort and 7 mice in BAY-D cohort and similar results were observed.

B. The effects of BAY-D or BAY-I treatment on tumor-associated immune cell landscape. Castrated *Pten*-null mice were treated with vehicle (n=23), BAY-D (n=7) or BAY-I (n=28) for 4 weeks, Tumor tissues were dissociated, numbers of tumor-associated immune cell populations were measured by FACS analysis. * $p < 0.05$, ** $p < 0.01$, *** $p < 0.001$ by two-sided T-test. Data were represented as mean$\pm$SEM.

C. BAY-I treatment does not influence $CD8^+$ T cells infiltration into other organs. Liver and lung from animals in (B) were fixed and sections were stained with anti-CD8 antibody, and $CD8^+$ T cell infiltrations were quantified. n=6 for each cohort. Data were presented as dot plots with mean as central lines; $p = 0.995$ for lung and $p = 0.386$ for liver by two-sided T-test.

D. BAY-I treatment induced $CD8^+$ T cells expansion and reduced Treg cells in primary prostate cancer. Intact *Pten*-null mice were treated with vehicle (n=11) or BAY-I (n=6) for 4 weeks. $CD45^+$ cell number, the percentage of $CD8^+$, $CD4^+$, Treg, $B220^+$ and MDSC in $CD45^+$, and $CD8^+$/Treg ratio were analyzed by combining FACS with cell counting and data were presented as dot plots with mean as central lines. * $p < 0.05$, ** $p < 0.01$, *** $p < 0.001$ by two-sided T-test. Source data and exact *p* values are provided in the Source Data file.

Supplementary Figure 5

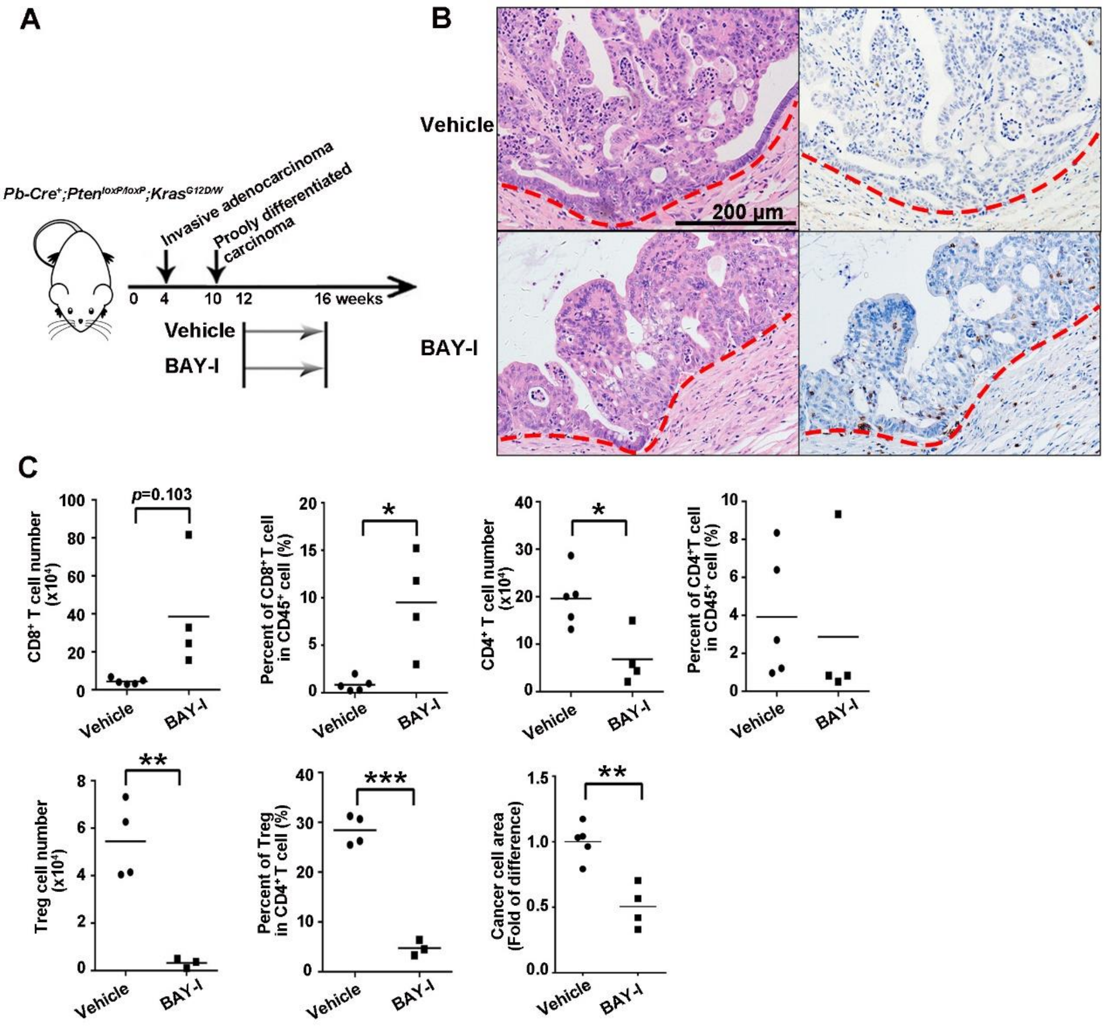

Supplementary Figure 5. The effects of BAY1082439 treatment on *Pb-Cre⁺;Pten$^{loxP/loxP}$;K-ras$^{G12D/W}$* (CPK) mice

The effects of BAY-I or BAY-D treatment on *Pb-Cre*$^{+}$*;Pten*$^{loxP/loxP}$*;K-ras*$^{G12D/W}$ mice. 12-week-old *Pb-Cre*$^{+}$*;Pten*$^{loxP/loxP}$*;K-ras*$^{G12D/W}$ mice were treated with vehicle (n=5) or BAY-I (n=4) for 4 weeks (A). Prostate tumors were isolated, one half of the tumor were fixed and stained with HE for cancer cell area quantification and anti-CD8 (B and C rightmost panel), The same staining was performed at 3 mice in each cohort and similar results were observed; the other half of the tumor tissues were dissociated, and the numbers of tumor-associated immune cell populations were measured by FACS (C). Data were

presented as dot plots with mean as central lines. * $p < 0.05$; ** $p < 0.01$, *** $p <0.001$ by two-sided T-test. Source data and exact *p* values are provided in the Source Data file.

Supplementary Figure 6

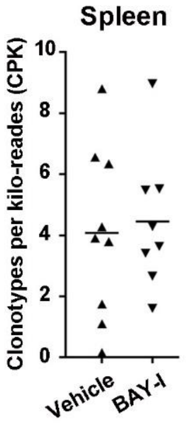

Supplementary Figure 6. BAY-I effect on spleen CD8$^+$ T cell clonotype diversity

BAY-I treatment do not alter spleen CD8$^+$T cell clonotype diversity. Castrated *Pten*-null mice were treated with vehicle (n=9) and BAY-I (n=8), spleen CD8$^+$ T cell were sorted by FACS, TCR clonotype diversities were calculated and presented as dot plots with mean as the central lines. Source data and exact *p* values are provided in the Source Data file.

Supplementary Figure 7

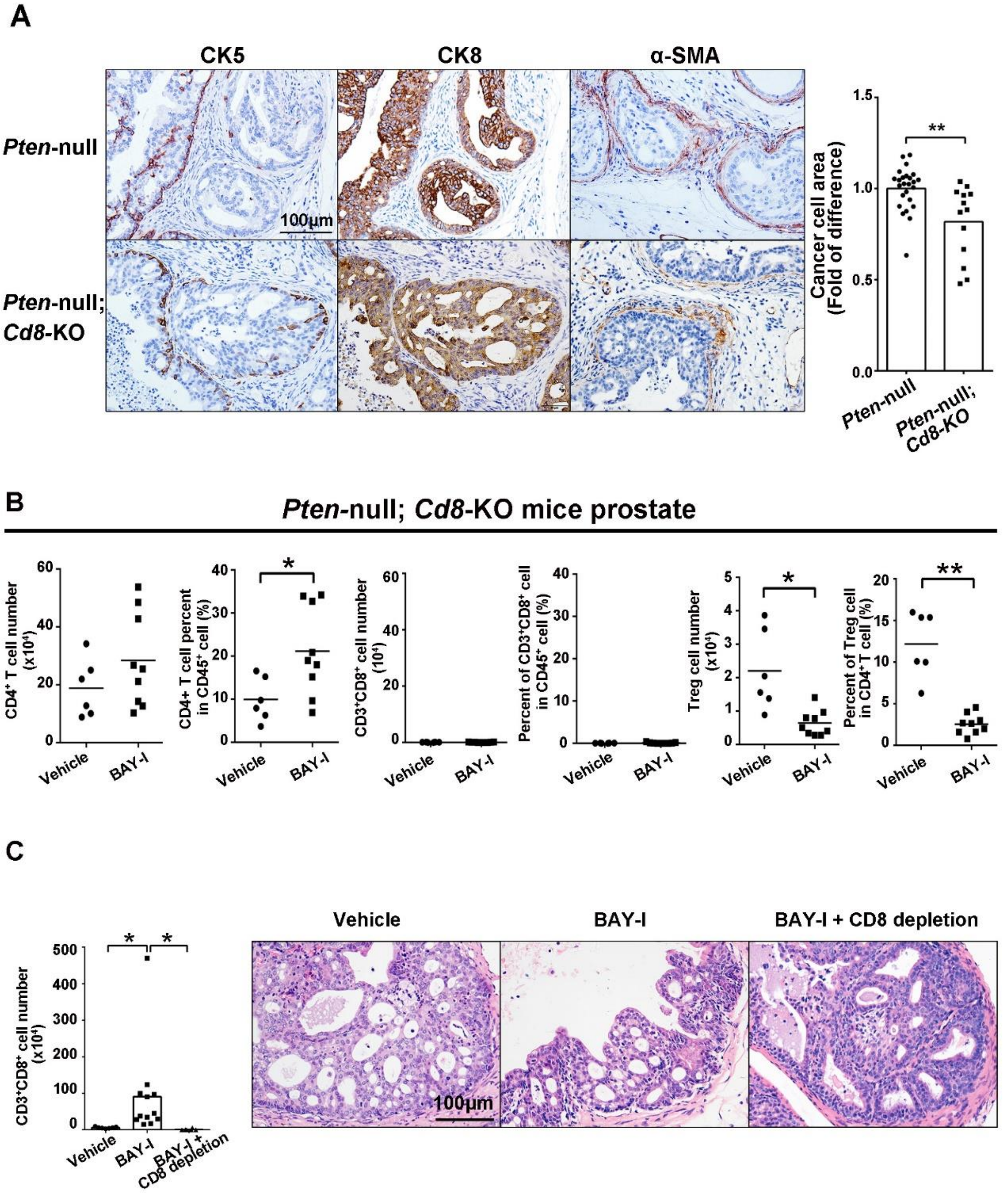

Supplementary Figure 7. The roles of CD8$^+$ T cell in BAY-I treatment induced immunity

A. *Cd8*-KO does not alter the kinetics of *Pten*-null prostate cancer development. 10 weeks old *Pten*-null and *Pten*-null;*Cd8*-KO mice (n>7 at both cohort) were treated with vehicle (for BAY-I) for 4 weeks, prostate tumors were harvested then stained with H&E,

anti-CK5/CK8 and α-SMA, and cancer cell areas in anterior lobes were measured and presented as dot plots with mean as central lines. **, *p* =0.0079 by two-sided T-test. For IHC, same staining were performed with 7 mice in each cohort and similar results were observed.

B. Effect of BAY-I treatment on *Pten*-null; *Cd8*-KO mice tumor immune populations. 10-week *Pten*-null; *Cd8*-KO mice were treated with vehicle (n=6) and BAY-I (n=9) for 4 weeks. $CD4^+$, $CD8^+$ and Treg cell numbers and the percentages of these cells in $CD45^+$ cells were analyzed by FACS and presented as dot plots with mean as central lines; * $p < 0.05$, ** $p < 0.01$ by two-sided T-test.

C. The effect of CD8 depletion on BAY-I treated castrated *Pten*-null prostate cancer model. Castrated *Pten*-null mice were treated with vehicle (n=7), BAY-I (n=12) or BAY-I plus anti-CD8 antibody (n=6) for 4 weeks. Prostate tumors were isolated, one half of the tumor tissues were dissociated, and the numbers of tumor-associated $CD8^+$T cell were measured by FACS analysis; the other half of the prostates were fixed and stained with HE. Data were presented as dot plots with mean as central lines. * $p < 0.05$ by two-sided T-test. For IHC, same staining were performed with 6 mice in vehicle and BAY-I plus anti-CD8 antibody cohort and 8 mice in BAY-I cohort and similar results were observed. Source data and exact *p* values are provided in the Source Data file.

Supplementary Figure 8

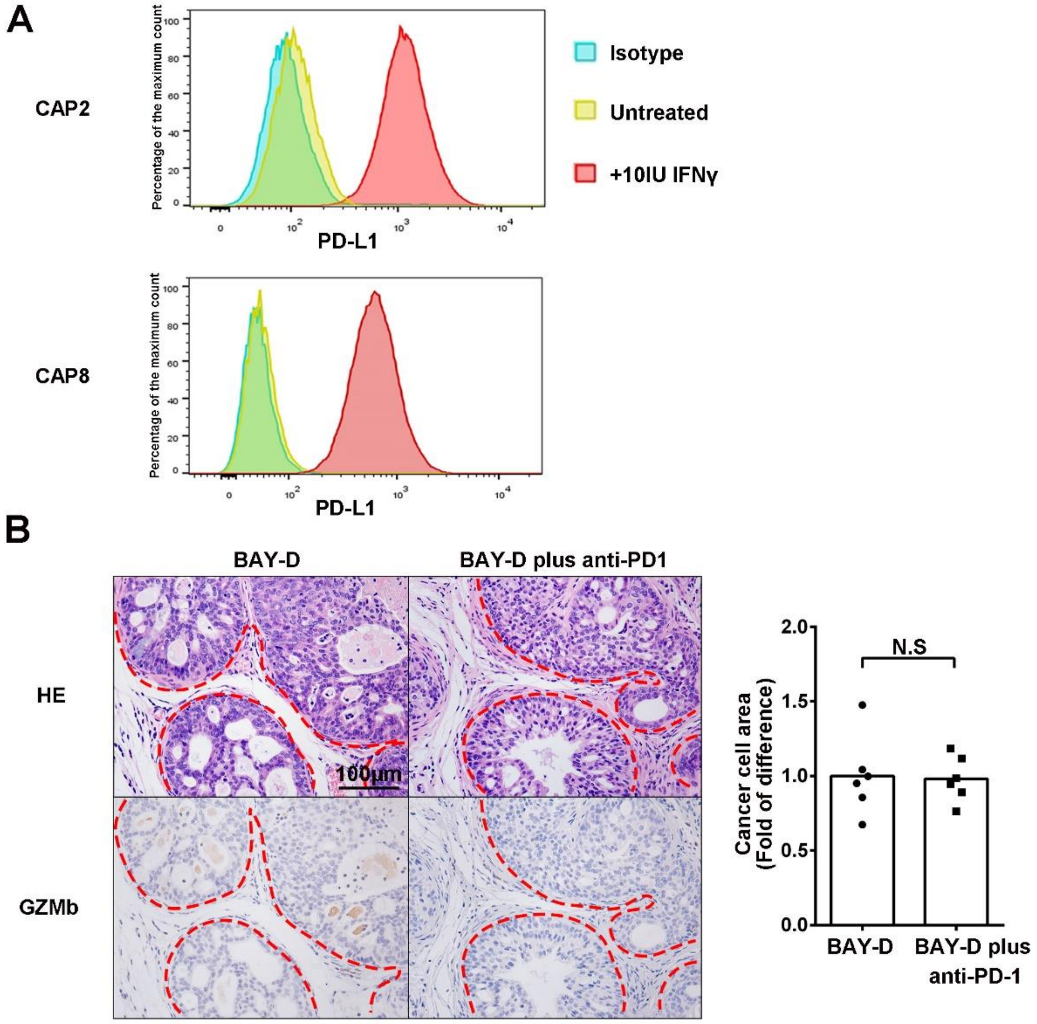

Supplementary Figure 8. IFNγ regulate PD-L1 expression in *Pten*-null cancer cell and BAY-D treatment combined with anti-PD-1.

A. IFNγ up-regulate PD-L1 expression in *Pten*-null prostate cancer cells. CAP2 and CAP8 cell lines were treated with or without IFNγ for 72h. PD-L1 expression levels were measured by FACS.

B. Therapeutic effects of BAY-D (n=6) and BAY-D plus anti-PD-1 (n=7) combination treatments evaluated by H&E and IHC analyses, cancer cell areas in anterior lobes were measured and were presented as dot plots with mean as central lines. N.S, *p* =

0.887 by two-sided T-test. For IHC, the same staining were performed with 6 mice in each cohort and similar results were observed. Source data and exact *p* values are provided in the Source Data file.

Supplementary Figure 9

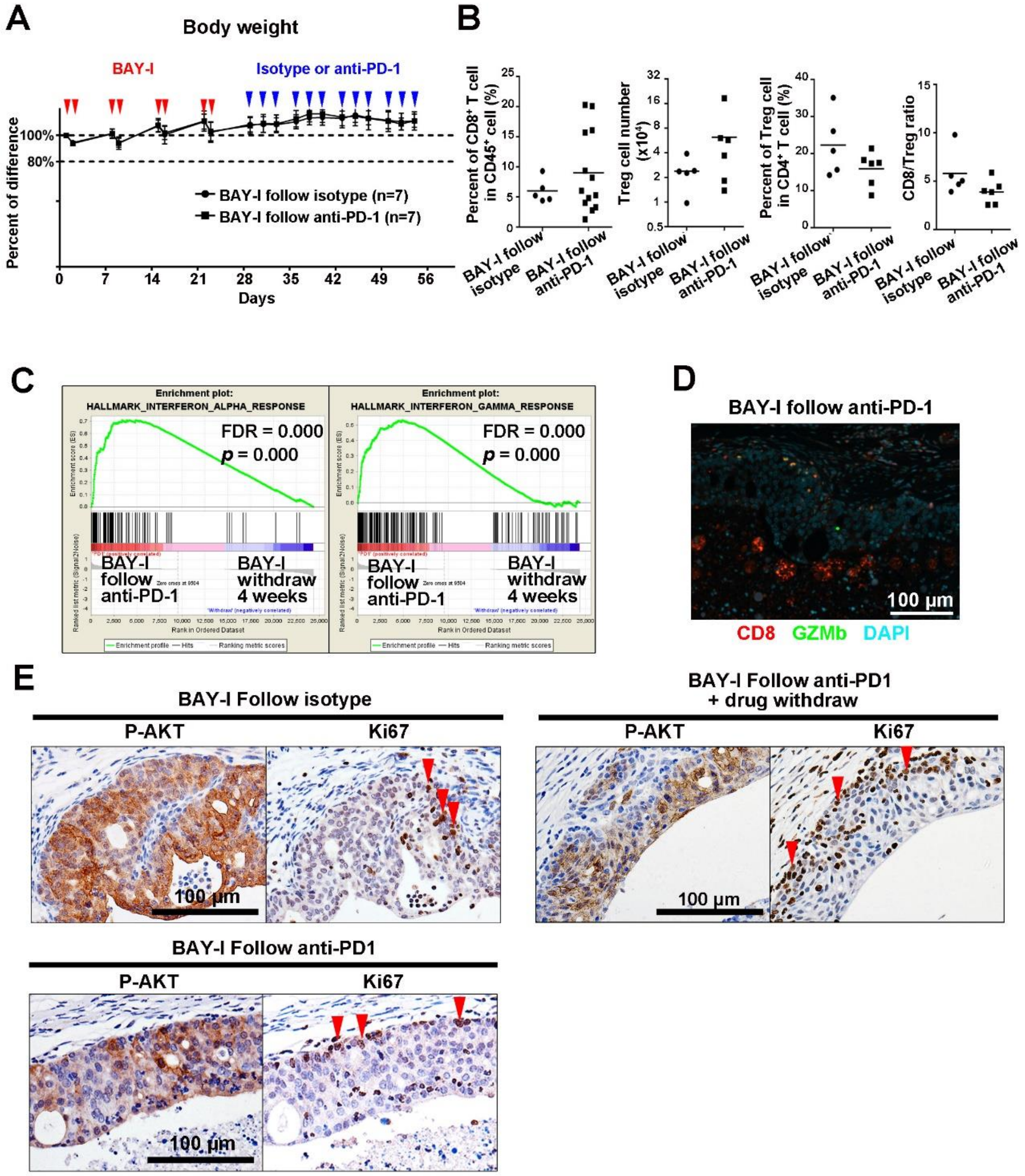

Supplementary Figure 9. Intermittent BAY1082439 treatment paves the way for subsequent anti-PD-1 therapy

A-B. The effect of sequential BAY-I and anti-PD-1 therapies on mouse body weight (n=7 at each cohort, presented by mean$\pm$SD) (A) and tumor-associated $CD8^+$ T and Treg cells, as compared to BAY-I monotherapy follow isotype, measured by FACS

analysis (n=5 at BAY-I follow isotype cohort and n=13 in BAY-I follow anti-PD-1 cohort) and were presented as dot plots with mean as central lines (B).

C. The effect of BAY-I follow anti-PD-1 on tumor tissue IFNα/γ pathway, as compared to BAY-I monotherapy withdraw group, by GSEA analysis. Statistical test was performed by GSEA.

D. $GZMb^{+}$;$CD8^{+}$ cells detected by co-immunofluorescence analysis in BAY-I follow anti-PD-1. The same staining were performed with 3 mice and similar results were observed.

E. Prostate tumor from BAY-I follow isotype cohort, BAY-I follow anti-PD-1 cohort and BAY-I follow anti-PD-1 drug withdraw cohort was stained with P-AKT and Ki67. Red arrow: $Ki67^{+}$ cancer cell. The same staining were performed with 6 mice in each cohort and similar results were observed. Source data and exact *p* values are provided in the Source Data file.

Supplementary Figure 10

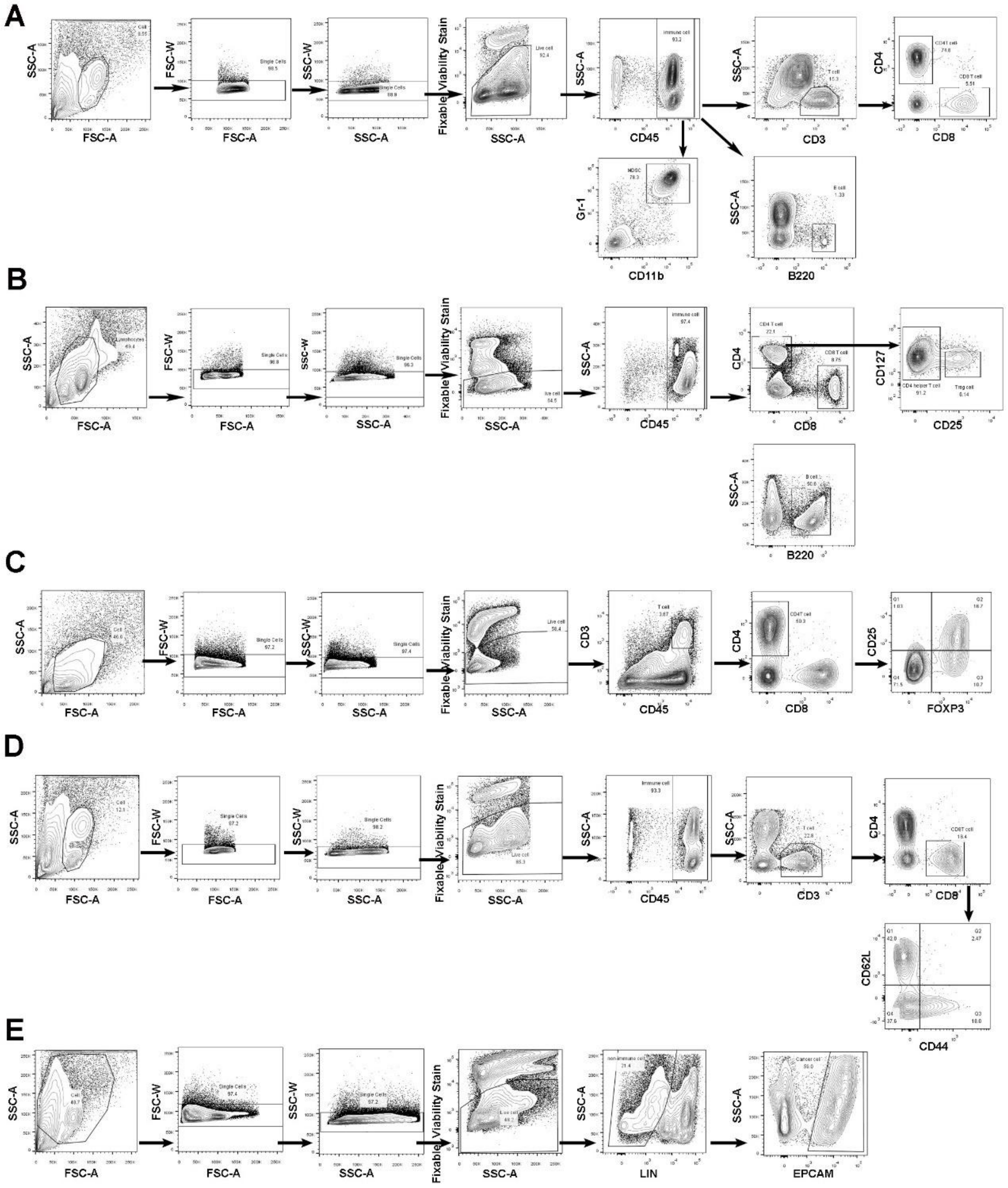

Supplementary Figure 10. FACS gating strategies for cell analysis or sorting

A. FACS gating strategy for analyzing immune cell landscape in Figure 2B, 4B-C, 6B, 8G and supplementary Figures 1C, 3A, 4B, 4D, 5C, 7B and 9B. Tumors were digested and stained with Fixable viability stain 450 for live cell, then stained with anti-CD45, CD3, CD4, CD8, B220, CD11b and Gr-1 antibodies. $CD8^{+}$T cell was defined as

$CD45^+CD3^+CD8^+$ cell, $CD4^+$T cell was defined as $CD45^+CD3^+CD4^+$ cell, B cell was defined as $CD45^+B220^+$ cell, MDSC cell was defined as $CD45^+CD11b^+GR\text{-}1^+$ cell.

B. FACS gating strategy for sorting and *in vitro* culture of $CD8^+$T cell, Helper T cell, Treg cell and B cell in Figure 3A. WT mice were executed and spleens were isolated, red blood cells were removed by using RBC lysis buffer, then the cells were stained with Fixable viability stain 450 for live cell and CFSE, then stained with anti-CD45, CD4, CD8, B220, CD25 and CD127 antibodies. $CD8^+$T cell was defined as $CD45^+CD8^+$ cell, Helper T cell was defined as $CD45^+CD4^+CD25^-$ cell, Treg cell was defined as $CD25^+CD4^+CD25^+CD127^{low/-}$ cell, B cell was defined as $CD45^+B220^+$ cell.

C. FACS gating strategy for analyzing regulatory T cell in Figure 3B-E and Treg analysis in supplementary Figures 4D, 5C, 7B, and 9B and $BrdU^+$CD8T cell at Figure 4D. For Treg analysis, tumors were digested and stained with Fixable viability stain 450 for live cell, then stained with anti-CD45, CD3, CD4, CD8 and CD25 antibodies, then stained with anti-FOXP3 using eBioscience™ FOXP3 Staining Buffer Set. $CD4^+$T cell was defined as $CD45^+CD3^+CD4^+$ cell, Treg cell was defined as $CD45^+CD3^+CD4^+$ $CD25^+FOXP3^+$ cell.

For BrdU positive cell staining, tumors were digested and stained with Fixable viability stain 450 for live cell, then stained with anti-CD45, CD3, CD4, CD8 antibodies, then $BrdU^+$cell was stained with anti-BrdU antibody by using Bioscience™ BrdU Staining Buffer Set. $BrdU^+$CD8T cell was defined as $CD45^+CD3^+CD8^+BrdU^+$ cell.

D. FACS gating strategy for analyzing naïve/memory phenotype or other markers in $CD8^+$ T cell in Figure 4D, 7E and 8A. Tumors were digested and stained with Fixable viability stain 450 for live cell, then stained with anti-CD45, CD3, CD4, CD8, then stained with CD44 and CD62L antibodies or other markers (PD-1, CTLA-4, Tim-3, ICOS and CD28 or corresponding isotype antibody). Naïve or effector memory $CD8^+$T cell were defined from $CD45^+CD3^+CD8^+$ population as $CD62L^+CD44^-$ or $CD44^+$ $CD62L^-$ cell.

E. FACS gating strategy for sorting of prostate cancer cell in Figure 8B or analyzing PD-L1 expression in Figure S8A. Castrated *Pten*-null mice were treated with BAY-I, and tumors were digested and stained with Fixable viability stain 450 for live cell, then stained with anti-Linage and EPCAM antibodies. Cancer cell was defined as $Linage^-$ $EPCAM^+$ cell.

For analysis PD-L1 expression in CAP2/8 cell, cancer cell line was digested by trypsin, then stained with Fixable viability stain 450 for live cell, then stained with PD-L1 antibody. Surface expression of PD-L1 in live cell were analyzed.

Supplementary Table 1. Primers used in this study.

Mouse primer	5‘ to 3'
mouse IRF1-F	TGCGCACCACTCCTTCGTCG
mouse IRF1-R	TGGCATGGTGGCTTTGCTGC
mouse IRF2-F	TCTCAGGCAAGCCGGGGACTA
mouse IRF2-R	TGCTTTCCTGTATGGATCGCCCAG
mouse IRF3-F	GGCTTGTGATGGTCAAGGTTGTTCC
mouse IRF3-R	TCCCTGTGCCTCCCTCCTGG
mouse IRF7-F	CAGCCAGCTCTCACCGAGCG
mouse IRF7-R	CCAGCTGAAGGCCGGGAGAC
mouse IRF9-F	CGGGGCGTAGTTCTGGGAGG
mouse IRF9-R	TGCCTGAGGCCATCCTTCTCCT
mouse IFITM1-F	GCTCCTCGACCACACCTCTTCAAA
mouse IFITM1-R	TTCAGGCACTTGGCGGTGGA
mouse IFIT1-F	AGCAACCATGGGAGAGAATGCTGA
mouse IFIT1-R	GCCAAGCTGCCCCTGTGGTA
mouse CCL5-F	CTCTGCCGCGGGTACCATGA
mouse CCL5-R	TCCTTCGAGTGACAAACACGACTGC
mouse CXCL10-F	GCCCACGTGTTGAGATCATTGCCA
mouse CXCL10-R	TGTGTGCGTGGCTTCACTCCA
Mouse IFNγ-F	TGGAGGAACTGGCAAAAGGATGGT
Mouse IFNγ-R	ATGCTTGGCGCTGGACCTGT
mouse B2M-F	TTCTGGTGCTTGTCTCACTGA
mouse B2M-R	CAGTATGTTCGGCTTCCCATTC
mouse βactin-F	CAGCCACTGTCGAGTCGCGT
mouse βactin-R	CACCATCACACCCTGGTGCCT
**Human primer**	**5‘ to 3'**

human IRF1-F	GGCCAACTTTCGCTGTGCCA
human IRF1-R	ACATGGCGACAGTGCTGGAGT
human IRF2-F	GAAGAGAGTGCCGAGGGGCG
human IRF2-R	CTGCTGCTGGATGCTGGGGT
human IRF3-F	ATCGTAGGCCGGACCATGGGA
human IRF3-R	CGGTTGAGGGCAGAGCGGAA
human IRF7-F	AGCTGTGCTGGCGAGAAGGC
human IRF7-R	CAGCACAGGCCCCAGTCAGG
human IRF9-F	GCCTGCGCTGTGCACTCAAC
human IRF9-R	CACTGGCCCCCTCCTCCTCATTA
human IFITM1-F	TCGCCTACTCCGTGAAGTCTAGGG
human IFITM1-R	AGGTTGCAGGCTATGGGCGG
human IFIT1-F	GGCAGACTGGCAGAAGCCCA
human IFIT1-R	CGGACAGCCTGCCTTAGGGG
human CCL5-F	AGACAGCACGTGGACCTCGC
human CCL5-R	TCGGGTGACAAAGACGACTGCT
human CXCL10-F	TCCTGCAAGCCAATTTTGTCCACG
human CXCL10-R	GGGAAGTGATGGGAGAGGCAGC
human B2M-F	GAGGCTATCCAGCGTACTCCA
human B2M-R	CGGCAGGCATACTCATCTTTT
human βactin-F	CATGTACGTTGCTATCCAGGC
human βactin-R	CTCCTTAATGTCACGCACGAT

Supplementary Table 2. Antibodies used in this study.

**Antibody used for FACS**		Dilution
Mouse CD45	biolegend 103147 / 103105 / 103108	1:100
Mouse CD11b	biolegend 101206	1:200
Mouse Gr-1	biolegend 108411	1:100
Mouse CD3	biolegend 100219	1:100
Mouse CD4	biolegend 100429	1:200
Mouse CD8	biolegend 100713	1:100
Mouse CD25	Thermo fisher 17-0251-82	1:100
Mouse B220	biolegend 103244	1:100
Mouse FOXP3	Thermo fisher 12-4771-82	1:100
Mouse Linage marker	biolegend 103113 102417 116221	1:100
Mouse Ep-Cam	biolegend 118213	1:100
Mouse PD-1	biolegend 135209	1:100
Mouse CTLA-4	biolegend 106305	1:100
Mouse Tim-3	biolegend 119721	1:100
Mouse CD28	biolegend 102105	1:100
Mouse ICOS	biolegend 107705	1:100
Mouse CD127	biolegend 135025	1:100
Mouse CD44	biolegend 103047	1:100
Mouse CD62L	biolegend 104405	1:200
Mouse PD-L1	biolegend 124311	1:100
Anti-BrdU-PE	Biolegend 339812	1:100
**Antibody used for IHC**		
Mouse CD8	98941, Cell Signaling Technology	1:200
Mouse granzyme-b	4059, Abcam	1:2000
Mouse Ki67	15580, Abcam	1:2000
anti-BrdU	6326, Abcam	1:200
Mouse P-AKT	4060, Cell Signaling Technology	1:200
Mouse CD11C	CST-97585	1:200
Mouse CK5	abcam52635	1:200
Mouse CK8	abcam53280	1:200
Mouse A-SMA	CST19245	1:200
Mouse AR	SANTA-sc-816	1:200
TRITC anti-Rabbit IgG	ZF-0316, ZSGB-BIO	1:100
FITC anti-rat IgG	ab7093, abcam	1:100

**Antibody used for Western bolt**		
Mouse/human actin	TA-09, Zsbio	1:1000
Mouse/human P-AKT （serine 473）	4060, Cell Signaling Technology	1:1000
Mouse/human Pan-AKT	4691, Cell Signaling Technology	1:1000
Mouse/human PTEN	9188, Cell Signaling Technology	1:1000
Mouse B2M	59035, Cell Signaling Technology	1:1000
HRP-conjugated anti-mouse antibody	115-035-003, Jackson ImmunoResearch Laboratories	1:5000
HRP-conjugated anti-rabbit antibody	111-035-003, Jackson ImmunoResearch Laboratories	1:5000
**Antibody used for In vivo treatment**		
InVivoPlus anti-mouse PD-1	BioXCell BE0146	
InVivoPlus rat IgG2a isotype control	BioXCell BE0089	
InVivoPlus anti-mouse CD8α	BioXCell BP0061	
**Antibody used for In vitro T cell culture**		
Ultra-LEAF™ Purified anti-mouse CD28 Antibody	Biolegend 102115	10ug/ml
Ultra-LEAF™ Purified anti-mouse CD3ε Antibody	Biolegend 100339	1ug/ml

Supplementary Table 3. The differential inhibitory effects of BAY1082439 and AKT, PI3Kβ , PI3Kδ on T cell and B cell populations.

IC50/umol	$CD8^+$ T cell	Helper T cell	Treg cell	B cell
BAY1082439	0.038	0.009	0.004	0.005
Ipatasertib(AKTi)	10.23	4.51	4.94	3.9
TGX-221(PI3Kβi)	10.06	6.26	3.04	0.195
CAL-101(PI3Kδi)	1.695	4.701	0.29	0.029

FACS sorted $CD8^+$, helper T, Treg and B cells from spleen of WT mice were cultured with different concentration of BAY1082439, AKT inhibitor, PI3Kβ inhibitor, and PI3Kδ inhibitor. The percentages of growth inhibition were calculated against vehicle controls and IC50s were calculated by GraphPad Prism 6. Source data are provided as a Source Data file
