# Supplementary material for: Overcoming resistance to immune checkpoint therapy in PTEN-null prostate cancer by intermittent anti-PI3Kα/β/δ treatment
Source: Nat Commun. 2022 Jan 10;13:182. doi: 10.1038/s41467-021-27833-0 (PMC8748754; doi:10.1038/s41467-021-27833-0)
Supplement: Supplementary file 2 — Reporting Summary [file 41467_2021_27833_MOESM2_ESM.pdf]

nature research

Corresponding author(s): Hong Wu

Last updated by author(s): Nov 30, 2021

Reporting Summary

Nature Research wishes to improve the reproducibility of the work that we publish. This form provides structure for consistency and transparency in reporting. For further information on Nature Research policies, see our Editorial Policies and the Editorial Policy Checklist.

Statistics

For all statistical analyses, confirm that the following items are present in the figure legend, table legend, main text, or Methods section.

n/a	Confirmed	
☐	☒	The exact sample size (n) for each experimental group/condition, given as a discrete number and unit of measurement
☐	☒	A statement on whether measurements were taken from distinct samples or whether the same sample was measured repeatedly
☐	☒	The statistical test(s) used AND whether they are one- or two-sided<br>*Only common tests should be described solely by name; describe more complex techniques in the Methods section.*
☒	☐	A description of all covariates tested
☐	☒	A description of any assumptions or corrections, such as tests of normality and adjustment for multiple comparisons
☐	☒	A full description of the statistical parameters including central tendency (e.g. means) or other basic estimates (e.g. regression coefficient) AND variation (e.g. standard deviation) or associated estimates of uncertainty (e.g. confidence intervals)
☐	☒	For null hypothesis testing, the test statistic (e.g. F, t, r) with confidence intervals, effect sizes, degrees of freedom and P value noted<br>*Give P values as exact values whenever suitable.*
☒	☐	For Bayesian analysis, information on the choice of priors and Markov chain Monte Carlo settings
☒	☐	For hierarchical and complex designs, identification of the appropriate level for tests and full reporting of outcomes
☒	☐	Estimates of effect sizes (e.g. Cohen's d, Pearson's r), indicating how they were calculated

Our web collection on statistics for biologists contains articles on many of the points above.

Software and code

Policy information about availability of computer code

Data collection	Real-Time PCR Analysis Software Bio-Rad CFX manager 3.1 was used for Real-Time PCR signal collection.<br>Image lab 5.0 was used for western blot signal collection.<br>BD FACSDiva 8.0 Software was used for FACS signal collection.<br>OLYMPUS cellSens standard software (version 1.11) was used for IHC image capture.
Data analysis	Real-Time PCR Analysis was performed by using Bio-Rad CFX manager 3.1 software.<br>GraphPad Prism 6 and Excel 2019 was used for data plotting and statistical analysis.<br>Image lab 5.0 was used for western blot data analyzing.<br>Flowjo V10 was used for FACS data analyzing.<br>Statistical analyses were conducted in Excel 2019.<br>Pathway activity score was calculated by R package GSVA V1.20.0.<br>Cancer cell area was determined by using ZEISS ZEN 2.3 software.<br>Tophat (version v2.1.1) was used to aligned RNAseq data to the mouse genome.<br>Cuffdiff (version v2.2.1) was used to identify differential expressed genes in RNAseq data.

For manuscripts utilizing custom algorithms or software that are central to the research but not yet described in published literature, software must be made available to editors and reviewers. We strongly encourage code deposition in a community repository (e.g. GitHub). See the Nature Research guidelines for submitting code & software for further information.

Data

Policy information about availability of data

All manuscripts must include a data availability statement. This statement should provide the following information, where applicable:

- Accession codes, unique identifiers, or web links for publicly available datasets
- A list of figures that have associated raw data
- A description of any restrictions on data availability

Source data are provided with this paper. All patient RNAseq data analyzed was available at TCGA database and from cBioPortal (https://www.cbioportal.org/). No restrictions on data availability. All RNAseq raw data used in Figure1, 2, 4, 6, 7, S2, S6, S9 and Supplementary data 1 was available at Gene Expression Omnibus under accession number GSE159660 (https://www.ncbi.nlm.nih.gov/geo/query/acc.cgi?acc=GSE159660). The remaining data are available within the Article, Supplementary Information or Source Data file.

Field-specific reporting

Please select the one below that is the best fit for your research. If you are not sure, read the appropriate sections before making your selection.

☒ Life sciences ☐ Behavioural & social sciences ☐ Ecological, evolutionary & environmental sciences

For a reference copy of the document with all sections, see nature.com/documents/nr-reporting-summary-flat.pdf

Life sciences study design

All studies must disclose on these points even when the disclosure is negative.

Sample size	Statistical methods were not used to predetermine sample size. Key findings were repeated in multiple independent experiments, as noted in figure legends. All sample size was at least three independent replicates. Animal and patient sample size was determined by experimental feasibility and sample availability to demonstrate certain results.
Data exclusions	No data were excluded from the analyses.
Replication	Biological replications (three biological replicates at least) and statistics were indicated in the legends. All attempts at replication were successful based on replications on different days showing comparable significance level for biological comparison.
Randomization	Samples were allocated into experimental groups by different drug treatments (Vehicle, or BAY1082439 treatment of different times) or by confirmed genetic modification of the cell line (doxycycline-induced protein expression). Mouse samples were randomly allocated into experimental groups after confirmed Pten genetic deletion.
Blinding	The researchers were blinded during animals research data collection, experiments apart from animal studies, and data analysis.

Reporting for specific materials, systems and methods

We require information from authors about some types of materials, experimental systems and methods used in many studies. Here, indicate whether each material, system or method listed is relevant to your study. If you are not sure if a list item applies to your research, read the appropriate section before selecting a response.

Materials & experimental systems

n/a	Involved in the study	
☐	☒	Antibodies
☐	☒	Eukaryotic cell lines
☒	☐	Palaeontology and archaeology
☐	☒	Animals and other organisms
☒	☐	Human research participants
☒	☐	Clinical data
☒	☐	Dual use research of concern

Methods

n/a	Involved in the study	
☒	☐	ChIP-seq
☐	☒	Flow cytometry
☒	☐	MRI-based neuroimaging

Antibodies

Antibodies used

anti-anti-Mouse CD45 for FACS	Biolegend 30-F11, #103147/#103105 1:100
anti-Mouse CD3 for FACS	Biolegend 17a2, #100219 1:100
anti-Mouse CD4 for FACS	Biolegend GK1.5, #100429 1:200
anti-Mouse CD8 for FACS	Biolegend 53-6.7,#100713 1:100
anti-Mouse CD25 for FACS	Thermo fisher PC61.5, #17-0251-82 1:100

anti-Mouse B220 for FACS	Biolegend RA3-6B2, #103244 1:100
anti-Mouse FOXP3 for FACS	Thermo fisher NRRF-30, #12-4771-82 1:100
anti-Mouse Linage marker for FACS	Biolegend 30-F11, #103113, 390, #102417, TER-119, #116221 1:100
anti-Mouse Ep-Cam for FACS	Biolegend G8.8, #118213 1:100
anti-Mouse PD-1 for FACS	Biolegend 29F.1A12, #135209 1:100
anti-Mouse CTLA-4 for FACS	Biolegend UC10-4B9,#106305 1:100
anti-Mouse Tim-3 for FACS	Biolegend RMT3-23,#119721 1:100
anti-Mouse CD28 for FACS	Biolegend HMβ1-1,#102215 1:100
anti-Mouse ICOS for FACS	Biolegend 15F9, #107705 1:100
anti-Mouse CD127 for FACS	Biolegend A7R34, #135025 1:100
anti-Mouse CD44 for FACS	Biolegend 1M7, #103047 1:100
anti-Mouse CD62L for FACS	Biolegend MEL-14, #104405 1:100
anti-Mouse PD-L1 for FACS	Biolegend 10F.9G2, #124311 1:100
anti-Mouse CD11b for FACS	Biolegend M1/70, #101206 1:200
anti-Mouse Gr-1 for FACS	Biolegend RB6-8C5, #108411 1:100
anti-Brdu for FACS	Biolegend Bu20a, #339812 1:100
anti-Mouse CD8 for IHC	Cell Signaling Technology D4W2Z, #98941 1:200
anti-Mouse CD11C for IHC	Cell Signaling Technology D1V9Y, #97585 1:200
anti-Mouse a-SMA for IHC	Cell Signaling Technology D4K9N, #19245 1:200
anti-Mouse granzyme-b for IHC (polyclonal)	Abcam #4059 1:1000
anti-Mouse Ki67 for IHC (polyclonal)	Abcam #15580 1:1000
anti-Mouse CK5 for IHC	Abcam EP1601Y, #52635 1:300
anti-Mouse CK8 for IHC	Abcam EP1628Y, #53280 1:300
anti-Mouse AR for IHC (polyclonal)	SANTA #sc-816 1:200
TRITC anti-Rabbit IgG for IF	ZSGB-BIO #ZF-0316 1:100
FITC anti-rat IgG for IF	Abcam #7093 1:100
anti-Brdu for IF	Abcam BU1/75 (ICR1), #6326 1:100
anti-Mouse/human actin for WB	Zsbio OTI1, #TA-09 1:1000
anti-Mouse/human P-AKT for WB	Cell Signaling Technology D9E, #4060 1:1000
anti-Mouse/human PTEN for WB	Cell Signaling Technology D4.3, #9188 1:1000
anti-Mouse B2M for WB (polyclonal)	Cell Signaling Technology #59035 1:1000
HRP-conjugated anti-mouse antibody for WB	Jackson ImmunoResearch Laboratories #115-035-003, 1:5000
HRP-conjugated anti-rabbit antibody for WB	Jackson ImmunoResearch Laboratories #111-035-003, 1:5000
Anti-mouse PD-1 for in vivo	BioXCell RMP1-14, #BE0146 200ug/dose
Isotype control for in vivo	BioXCell 2A3, #BE0089 200ug/dose
Anti-mouse CD8a for in vivo	BioXCell 2.43, #BP0061 200ug/dose
Ultra-LEAF™ Purified anti-mouse CD3ε Antibody for T cell culture	Biolegend 145-2C11, #100339 10ug/ml
Ultra-LEAF™ Purified anti-mouse CD28 Antibody for T cell culture	Biolegend 37.51, #102115 1ug/ml

Validation

All western blot and flow cytometry antibodies in the manuscript had been validated. Each primary antibody data provided in the manuscript has been validated for the species and application on the manufacturer's website.

anti-anti-Mouse CD45 for FACS Biolegend 30-F11, #103147/#103105 was successfully stained in C57BL/6 mouse splenocytes.

anti-Mouse CD3 for FACS Biolegend 17a2, #100219 was successfully stained in C57BL/6 mouse splenocytes.

anti-Mouse CD4 for FACS Biolegend GK1.5, #100429 was successfully stained in C57BL/6 mouse splenocytes.

anti-Mouse CD8 for FACS Biolegend 53-6.7,#100713 was successfully stained in C57BL/6 mouse splenocytes.

anti-Mouse CD25 for FACS Thermo fisher PC61.5, #17-0251-82 was successfully stained in SJL mouse splenocytes.

anti-Mouse B220 for FACS Biolegend RA3-6B2, #103244 was successfully stained in C57BL/6 mouse splenocytes.

anti-Mouse FOXP3 for FACS Thermo fisher NRRF-30, #12-4771-82 was successfully stained in BALB/c mouse splenocytes.

anti-Mouse Linage marker for FACS Biolegend 30-F11, #103113, 390, #102417, TER-119, #116221 was successfully stained in C57BL/6 mouse splenocytes.

anti-Mouse Ep-Cam for FACS Biolegend G8.8, #118213 was successfully stained in TE-71 (mouse thymic epithelial stromal cell line).

anti-Mouse PD-1 for FACS Biolegend 29F.1A12, #135209 was successfully stained in IL-2 stimulated C57BL/6 mouse splenocytes.

anti-Mouse CTLA-4 for FACS Biolegend UC10-4B9,#106305 was successfully stained in IL-2 stimulated C57BL/6 mouse

splenocytes.

anti-Mouse Tim-3 for FACS Biolegend RMT3-23,#119721 was successfully stained in Mouse Tim-3 transfected cells.

anti-Mouse CD28 for FACS Biolegend HMβ1-1,#102105 was successfully stained in C57BL/6 mouse splenocytes.

anti-Mouse ICOS for FACS Biolegend 15F9, #107705 was successfully stained in Con-A stimulated (3 days) BALB/c splenocytes.

anti-Mouse CD127 for FACS Biolegend A7R34, #135025 was successfully stained in C57BL/6 mouse splenocytes.

anti-Mouse CD44 for FACS Biolegend 1M7, #103047 was successfully stained in C57BL/6 mouse splenocytes.

anti-Mouse CD62L for FACS Biolegend MEL-14, #104405 was successfully stained in C57BL/6 mouse bone marrow cells.

anti-Mouse PD-L1 for FACS Biolegend 10F.9G2, #124311 was successfully stained in C57BL/6 mouse splenocytes.

anti-Mouse CD11b for FACS Biolegend M1/70, #101206 was successfully stained in C57BL/6 mouse bone marrow cells.

anti-Mouse Gr-1 for FACS Biolegend RB6-8C5, #108411 was successfully stained in C57BL/6 mouse bone marrow cells.

anti-Brdu for FACS Biolegend Bu20a, #339812 was successfully stained in Brdu-incorporated Hut-78 cells.

anti-Mouse CD8 for IHC Cell Signaling Technology D4W2Z, #98941 was successfully stained in paraffin-embedded mouse spleen tissue section.

anti-Mouse CD11C for IHC Cell Signaling Technology D1V9Y, #97585 was successfully stained in paraffin-embedded 4T1 mammary tumor tissue section.

anti-Mouse a-SMA for IHC Cell Signaling Technology D4K9N, #19245 was successfully stained in mouse skeletal muscle tissue section.

anti-Mouse granzyme-b for IHC (polyclonal) Abcam #4059 was successfully stained in mouse GBM xenograft tissue section.

anti-Mouse Ki67 for IHC (polyclonal) Abcam #15580 was successfully stained in mouse spleen tissue section.

anti-Mouse CK5 for IHC Abcam EP1601Y, #52635 was successfully stained in mouse skin tissue section.

anti-Mouse CK8 for IHC Abcam EP1628Y, #53280 was successfully stained in mouse liver tissue sections.

anti-Mouse AR for IHC (polyclonal) SANTA #sc-816 (302 citations) was successfully stained in MDA-MB-468 cells (https://pubmed.ncbi.nlm.nih.gov/26976651/).

TRITC anti-Rabbit IgG for IF ZSGB-BIO #ZF-0316 was showing specifiably binding with Rabbit IgG but no other species.

FITC anti-rat IgG for IF Abcam #7093 was successfully stained in NOD/SCID mice kidney sections (https://pubmed.ncbi.nlm.nih.gov/34079330/).

anti-Brdu for IF Abcam BU1/75 (ICR1), #6326 was successfully stained in Brdu treated Hela cell.

anti-Mouse/human actin for WB Zsbio OTI1, #TA-09 was successfully stained in Hela cell line and other 6 cell lines.

anti-Mouse/human P-AKT for WB Cell Signaling Technology D9E, #4060 was successfully stained in paraffin-embedded MDA-MB-468 xenograft tissue section and sucessfully detected in extracts of PC-3 cell line.

anti-Mouse/human PTEN for WB Cell Signaling Technology D4.3, #9188 was successfully detected from extracts from various human cell lines

anti-Mouse B2M for WB (polyclonal) Cell Signaling Technology #59035 was successfully detected from extracts from various mouse cell lines.

HRP-conjugated anti-mouse antibody for Jackson ImmunoResearch Laboratories #115-035-003, showing successfully reacts with whole molecule mouse IgG by immunoelectrophoresis or ELISA

HRP-conjugated anti-rabbit antibody for Jackson ImmunoResearch Laboratories #111-035-003, showing successfully reacts with whole molecule rabbit IgG by immunoelectrophoresis or ELISA

Anti-mouse PD-1 for in vivo BioXCell RMP1-14, #BE0146: 15 citations, reported applications include in vivo PD-1 blockade.

Isotype control for in vivo BioXCell 2A3, #BE0089 BioXCell RMP1-14, #BP0146: 11 citations, reported applications include control antibody for in vivo PD-1 blockade.

Anti-mouse CD8a for in vivo BioXCell 2.43, #BP0061 website validation: 17 citations, reported applications include in vivo CD8T cell depletion.

Ultra-LEAF™ Purified anti-mouse CD3ε Antibody for T cell culture Biolegend 145-2C11, #100339: 21 citations, reported applications include in vitro T cell activation assays.

Ultra-LEAF™ Purified anti-mouse CD28 Antibody for T cell culture Biolegend 37.51, #102115: 16 citations, reported applications include in vitro costimulation of T and NK cells.

Eukaryotic cell lines

Policy information about cell lines

Cell line source(s)	Human prostate cancer cells (PC3 and LNCAP) were ordered from ATCC. Mouse prostate cancer cells (CAP2 and CAP8) were established by our lab (Jing Jiao. et al. Cancer research, 2007). PC3 WT/PTEN-inducible cells were established by our lab (David J Mulholland. et al. Cancer cell, 2011).
Authentication	PC3 and LNCAP cell line have not been authenticated after purchasing from vendors. CAP2 and CAP8 cell were authenticated by PCR-based genotyping analysis. PC3 WT/PTEN-inducible cells were authenticated by immunoblotting of targeted proteins and Sanger sequencing of modification region of targeted genes.
Mycoplasma contamination	All cells used have been tested negative for mycoplasma contamination.
Commonly misidentified lines (See ICLAC register)	No such cell lines were used.

Animals and other organisms

Policy information about studies involving animals; ARRIVE guidelines recommended for reporting animal research

Laboratory animals	The generation of the Pb-Cre+;PtenloxP/loxP prostate cancer model (male, 10 weeks) has been described previously (Wang, S. et al. Cancer cell, 2003). Cd8atm1Mak mice (CD8KO mice) was purchased from the Jackson lab (002665) then crossed with the Pten-null mice to generate Pb-Cre+;PtenL/L;Cd8-/- (Pten-null,Cd8-Ko mice. male, 10 weeks) mice. The generation of the Pb-Cre+;PtenL/L;K-rasG12D/W prostate cancer model (male, 12 weeks) has been described previously (Mulholland DJ. et al. Cancer research, 2012). Animal housing, breeding, and surgical procedures were approved by the Ethics Committee under ID LSC-WuH-1 and conducted in accordance with the regulations of the Division of Laboratory Animal Medicine at Peking University. Animals were housed at 22°C, with humidity of 40-70%, and dark/light cycle of 12/12 hours. For all animal experiments, the animals were monitored carefully, and no body-weight loss exceeds 20% in all treatment cohorts.
Wild animals	The study did not involve wild animals.
Field-collected samples	The study did not involve field-collected samples
Ethics oversight	Animal housing, breeding, and surgical procedures were approved by the Ethics Committee under ID LSC-WuH-1 and conducted in accordance with the regulations of the Division of Laboratory Animal Medicine at Peking University.

Note that full information on the approval of the study protocol must also be provided in the manuscript.

Flow Cytometry

Plots

Confirm that:

- [x] The axis labels state the marker and fluorochrome used (e.g. CD4-FITC).
- [x] The axis scales are clearly visible. Include numbers along axes only for bottom left plot of group (a 'group' is an analysis of identical markers).
- [x] All plots are contour plots with outliers or pseudocolor plots.
- [x] A numerical value for number of cells or percentage (with statistics) is provided.

Methodology

Sample preparation	Cell were digested by 0.25% trypin, mouse prostates were minced in sterile tissue culture dishes, and subjected to collagenase A (1.5 mg/ml; Roche) and DNase I (0.1 mg/ml; Roche) digestion for 1 h at 37°C with constant agitation. single cell were re-suspended in PBS containing 1% FBS.
Instrument	For cell sorting, Cell were sorted on BD FACSAria™ III Cell Sorter.<br>For cell population ratio/protein expression analysis, cell was analyzed in BD LSRFortessa™ Flow Cytometer.

Software	BD FACSuite Flow Cytometry Software was used for FACS signal collection.<br>Flowjo V10 was used for FACS data analyzing.
Cell population abundance	Cell purity was >99% determined by post-sort purity checks of representative samples.
Gating strategy	cells were first gated using FSC/SSC characteristics, then doublets were sequentially excluded by combining FSC/SSC height and area signals. Dead cell was excluded by using BD Fixable Viability Stain 450 (562247).

☒ Tick this box to confirm that a figure exemplifying the gating strategy is provided in the Supplementary Information.
