# Supplementary material for: Overcoming resistance to immune checkpoint therapy in PTEN-null prostate cancer by intermittent anti-PI3Kα/β/δ treatment
Source: Nat Commun. 2022 Jan 10;13:182. doi: 10.1038/s41467-021-27833-0 (PMC8748754; doi:10.1038/s41467-021-27833-0)
Supplement: Supplementary file 3 — Description of Additional Supplementary Files [file 41467_2021_27833_MOESM3_ESM.pdf]

Title: Supplementary Data 1.
Description: Lists of Pathway and gene expression alterations detected by RNAseq analysis.
